# Single-nucleus CUT&RUN elucidates the function of intrinsic and genomics-driven epigenetic heterogeneity in head and neck cancer progression

**DOI:** 10.1101/gr.279105.124

**Published:** 2025-01

**Authors:** Howard J. Womersley, Daniel Muliaditan, Ramanuj DasGupta, Lih Feng Cheow

**Affiliations:** 1 Institute for Health Innovation and Technology, National University of Singapore, Singapore 117599, Singapore;; 2 Department of Biomedical Engineering, Faculty of Engineering, National University of Singapore, Singapore 117583, Singapore;; 3 Genome Institute of Singapore (GIS), Agency for Science, Technology and Research (A*STAR), Singapore 138672, Singapore

## Abstract

Interrogating regulatory epigenetic alterations during tumor progression at the resolution of single cells has remained an understudied area of research. Here we developed a highly sensitive single-nucleus CUT&RUN (snCUT&RUN) assay to profile histone modifications in isogenic primary, metastatic, and cisplatin-resistant head and neck squamous cell carcinoma (HNSCC) patient–derived tumor cell lines. We find that the epigenome can be involved in diverse modes to contribute toward HNSCC progression. First, we demonstrate that gene expression changes during HNSCC progression can be comodulated by alterations in both copy number and chromatin activity, driving epigenetic rewiring of cell states. Furthermore, intratumor epigenetic heterogeneity (ITeH) may predispose subclonal populations within the primary tumor to adapt to selective pressures and foster the acquisition of malignant characteristics. In conclusion, snCUT&RUN serves as a valuable addition to the existing toolkit of single-cell epigenomic assays and can be used to dissect the functionality of the epigenome during cancer progression.

Tumor metastasis and acquired drug resistance are key steps in cancer progression that ultimately lead to treatment failure and patient mortality. To identify novel modalities to prevent cancer progression and improve patient survival, it is important to understand the underlying mechanisms that drive these processes. To date, efforts to comprehend the basis of cancer progression have largely focused on uncovering genetic alterations within a tumor population that exert selective fitness, which allows cells to survive during drug treatment. However, cancers can exhibit marked cell-to-cell variation (intra-tumor heterogeneity [ITH]) in gene expression and their functional phenotypes that cannot always be explained by mutations or structural variations in their DNA. Nongenetic basis for ITH suggests a potential role for epigenetic mechanisms driving transcriptomic/phenotypic heterogeneity (Brock et al. 2009). Epigenetic alterations offer a heritable mechanism for generating ITH (Easwaran et al. 2014) and occur at greater frequencies in human cancers than in genetic mutations (Guo et al. 2019), thereby underscoring the importance of interrogating them when mapping trajectories of cellular plasticity–driven tumor evolution to metastatic or treatment-resistant disease. Cancer cells that survive sublethal challenges can often activate stress response pathways that confer early developmental, stem-like features that enable them to cope with further insults (Pisco and Huang 2015). These prosurvival alterations are often manifested in the aberrant modification of histone proteins (Füllgrabe et al. 2011). Furthermore, dysfunction in histone-modifying enzymes has been shown to have a causal relationship with cancer initiation and progression (Wang et al. 2016). Thus, understanding the underlying epigenetic mechanisms that underpin the evolution of cancer is crucial for the discovery of alternative therapeutic interventions to halt or delay its progression and to improve the survival of cancer patients in the clinic.

One such cancer type in which epigenetic ITH remains an understudied area of research is head and neck squamous cell carcinoma (HNSCC). Single-cell studies on HNSCC mainly focused on transcriptomic ITH, underscoring the need to investigate epigenetic control of ITH in HNSCC (Puram et al. 2017; Qi et al. 2021; Choi et al. 2023; Quah et al. 2023). An area in which epigenetic ITH can have a role in HNSCC progression is in the alterations of histone modifications. Histone modifications can occur rapidly as cells respond and adapt to the environment. Classically these changes are identified by chromatin immunoprecipitation followed by sequencing (ChIP-seq). However, classical ChIP-seq is only suitable for bulk cell assays, which have limited application for discerning epigenetically distinct subpopulations within tumors. This makes it difficult to determine whether plasticity or lineage transitions are a result of Darwinian selection of rare, pre-existing clones, or adaptation, in which dynamic epigenetic changes may activate distinct transcriptomic programs that lead to the emergence of new phenotypes. Thus, there is a need for studies employing methodologies that can detect histone modifications at the resolution of single cells in order to answer questions related to how epigenetic heterogeneity and plasticity may drive cell state transitions during HNSCC progression. Given that many readers and writers of different histone marks are amenable to pharmacological interventions (Helin and Dhanak 2013; Kakiuchi et al. 2021), a greater understanding of their regulatory function during tumor progression could result in the identification of new therapeutic intervention strategies for HNSCC patients.

Methods for profiling histone modifications or transcription factors (TFs) in single cells generally involve immunoprecipitation, using either an antibody immobilized nuclease or an antibody immobilized transposase, as exemplified by scChIP-seq, uliCUT&RUN, and scCUT&Tag, respectively (Rotem et al. 2015; Hainer et al. 2019; Kaya-Okur et al. 2019). Because of the paucity of DNA within single cells and the implicit requirement for selection of only a tiny fraction of this material, these methods and others tend to have low numbers of filtered reads and/or poor specificity. Single-cell ChIP-seq methods employ microfluidic devices to compartmentalize single cells, and although thousands of cells can be analyzed simultaneously, the number of unique reads per cell tends to be low owing to inefficient reactions within individual droplets. Additionally, the requirement for special equipment limits the adoption of this approach by the wider scientific community. On the other hand, the use of immobilized transposases has generated significantly more traction (Ai et al. 2019; Carter et al. 2019; Wang et al. 2019), largely because these methods do not require a library preparation stage that normally leads to additional loss of already scarce material. However, considerable off-target transposase binding occurs without stringent salt washes, which can inadvertently detach proteins of interest from DNA unless they are tightly bound, such as the case with TFs (Kaya-Okur et al. 2020). CUT&RUN was developed as an alternative to ChIP-seq that exhibits significantly less background noise (Skene and Henikoff 2017), which enables it to profile as few as a 100 cells (Skene et al. 2018). A single-cell version, uliCUT&RUN, was demonstrated to localize NANOG and SOX2 TFs in rare populations of mouse embryonic stem cells (Hainer et al. 2019). However, the extensive sequencing depth required for this method (median raw read number of 10,189,952 and 14,717,791 reads for single-cell uliCUT&RUN and bulk uliCUT&RUN, respectively) makes it impractical for analyzing large numbers of single cells (Hainer et al. 2019; Patty and Hainer 2021).

Given the challenges affecting other single-cell histone modification profiling methods such as uliCUT&RUN, we aimed to develop an improved nuclease-based assay to profile histone modifications in single nuclei. Here we developed single-nucleus CUT&RUN (snCUT&RUN) in order to gain more insights in the role of the epigenome and epigenetic heterogeneity during HNSCC progression. Altogether, we demonstrate that high-resolution profiling of histone modifications in single cells with snCUT&RUN can yield valuable insights into epigenetic heterogeneity in HNSCC, complementing existing single-cell transcriptomic studies.

## Results

### Development and performance of snCUT&RUN

Tagmentation-based methods for single-cell histone modification profiling (e.g., scCUT&Tag) have limitations with efficiency for the following reasons: (1) two independent transposition reactions are needed to create a sequenceable fragment, and (2) productive reactions require that the adapters introduced by transposition on each end of the fragment to be of different “types,” which only happens with 50% probability. Considering these challenges, we focused on the nuclease-based protocol: CUT&RUN. Unlike transposon-based methods, which get expended during the reaction, a single pA-MN enzyme in CUT&RUN can catalyze fragmentation of DNA on both sides of a given nucleosome. This means that after digestion, all fragments have the potential to yield sequenceable products after the library preparation stage. Although the library preparation protocol is more complex compared with that of scCUT&Tag, we believe that the increased sensitivity of snCUT&RUN would better cater to the needs for applications that require higher-sensitivity histone profiling at single-cell resolution.

**Figure 1.**
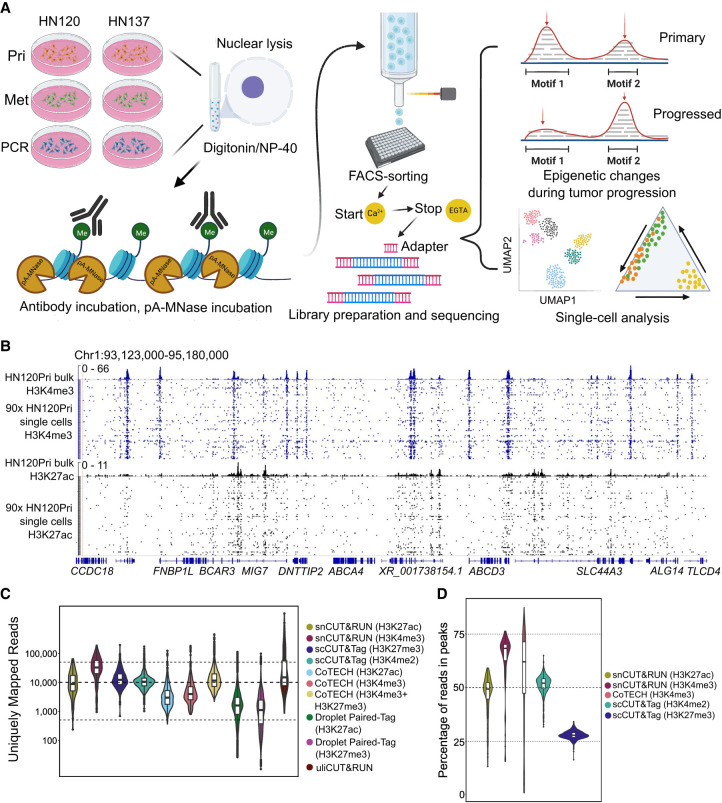
Schematic representation of overall workflow and quality control of snCUT&RUN. (*A*) Schematic overview of snCUT&RUN, applied on matched primary, metastatic, and primary cisplatin-resistant patient-derived head and neck cancer cell lines from two patients (HN120 and HN137). Image created with bioRender (https://www.biorender.com/) under publication license HI248I94WF. (*B*) Representative IGV-track image showing H3K4me3 and H3K27ac single-cell profiles of 90 HN120Pri cells, with corresponding bulk cell data for each mark (Robinson et al. 2011). (*C*) Violin- and boxplots showing the distribution of the number of unique mapped reads (UMRs) for each single-cell, for both H3K4me3 (median = 32,666) and H3K27ac (median = 8766). Dotted lines are at 50,000, 10,000, and 500 UMRs. snCUT&RUN data were benchmarked against uliCUT&RUN (Hainer et al. 2019), scCUT&Tag (Kaya-Okur et al. 2019), CoTECH (Xiong et al. 2021), and Droplet Paired-Tag (Xie et al. 2023). (*D*) Violin- and boxplots illustrating the percentage of reads in peaks for each single cell. H3K4me3 median = 68%, H3K27ac median = 49%. Dotted lines are at 75%, 50%, and 25%. snCUT&RUN was compared with CoTECH and scCUT&Tag.

A schematic of snCUT&RUN is shown in Figure 1A, and the detailed protocol is provided in the Methods section. The original CUT&RUN method has low background noise and requires a fraction of the sequencing depth compared with ChIP-seq. We found that several enhancements could transform this technique into a single-cell method that could be performed in a standard molecular biology laboratory with access to a FACS facility (or other means of single-cell isolation). First, we found that it was imperative to maintain nuclear integrity throughout the entire workflow to minimize background reads and ensure high-quality data. Damage to nuclei results in release of ambient DNA, clumping of nuclei, and loss of material. Hence, we formulated a lysis buffer with minimal detergent concentration, and the addition of sucrose supplemented with BSA resulted in lysis with vastly reduced clumping when resuspending pellets during washing steps throughout the workflow. Lysis, antibody, and pA-MN binding steps were all performed in bulk, which reduced the amount of handling and the loss of individual cells after isolation. Second, directly after pA-MN incubation, the nuclei were stained in buffer containing a nuclear dye and a labelled antinuclear pore complex antibody. This step greatly improved the efficiency in isolating single intact nuclei rather than multiple nuclei or debris. Third, low-salt conditions have been shown to prevent pA-MN from diffusing after digestion, thereby reducing off-target cleavage of background DNA (Meers et al. 2019). We adopted this approach by directly sorting single nuclei into a low-salt calcium buffer to initiate nuclease activity. Lastly, given that the library preparation stage is the major source of sample loss, the inactivation of pA-MN, end-repair, A-tailing, indexed adapter ligation, and SPRI steps were all performed with no tube transfers. The amount of paramagnetic SPRI beads used to remove unbound adapters was doubled relative to PEG/NaCl to increase the surface area available for binding to the DNA template. Furthermore, 50N neodymium magnets were used for separation to minimize dead volumes. By avoiding Proteinase K treatment, intact nuclei and high-molecular-weight debris could be observed being retained by the SPRI beads after eluting the target DNA, thereby reducing background. PCR was performed on pooled samples, the products concentrated via precipitation, and adapter monomers and dimers removed with a second set of SPRI bead treatment in preparation for high-throughput sequencing (Methods).

We first assessed the technical performance of snCUT&RUN on 94 sorted nuclei, probed with either anti-H3K4me3 or anti-H3K27ac antibodies. The sequencing read distribution of single cells exhibited a high degree of similarity with bulk cell populations probed with the respective histone-specific antibodies (Fig. 1B), demonstrating excellent specificity of our protocol. H3K4me3 is a histone mark that is generally associated with promoters. The read density profile from H3K4me3 snCUT&RUN samples showed similar profiles around transcriptional start sites (TSSs) to that of the bulk cell set, with corresponding patterns of nucleosome depletion (Supplemental Fig. S1A), indicating the high resolution of this assay. We next benchmarked snCUT&RUN with currently available single-cell histone modification profiling methods uliCUT&RUN (Hainer et al. 2019), scCUT&Tag (Kaya-Okur et al. 2019), CoTECH (Xiong et al. 2021), and Droplet Paired-Tag (Xie et al. 2023), using several quality-control (QC) metrics such as the number of uniquely mapped reads (UMRs) and fraction of reads in peaks (FRiP). The median number of unique reads for H3K4me3 was 32,666 (median number of unique reads mapping to annotated promoters = 6828 or 21.4% of total unique reads), whereas the corresponding number of unique reads for H3K27ac was 8766 (Fig. 1C). This was comparable or greater than the other methods tested. As an indication of signal to noise, the median FRiP was 68% for H3K4me3 and 49% for H3K27ac, which is likewise on par with or greater than other single-cell histone assays (Fig. 1D).

Comparison of the histone profiles of pooled snCUT&RUN with bulk CUT&RUN yielded a Pearson's correlation 0.956 and 0.6089 for H3K4me3 and H3K27ac, respectively (Supplemental Fig. S1B,C). Collectively, these data indicated that snCUT&RUN recapitulated bulk CUT&RUN data to a high degree and that this method has the potential to resolve subtle epigenetic differences that might be expected within cancer cell populations. Once more than 1000 cells had been screened for H3K4me3 and H3K27ac, we compared the performance of snCUT&RUN with the other, previously mentioned available methods by ranking the cells by number of UMRs (Supplemental Fig. S1D). Although in some cases different antibodies were used, we reasoned that there should be considerable overlap between the profiles of some modifications such as H3K4me2 and H3K4me3, being present at transcribing, and poised plus transcribing genes, respectively (Hyun et al. 2017). Under our experimental conditions, we could achieve almost an order of magnitude more UMRs than scCUT&Tag, even after downsampling raw snCUT&RUN read pairs by 25% to match scCUT&Tag raw read pair numbers. As a second metric, we compared the downsampled snCUT&RUN with scCUT&Tag and uliCUT&RUN by determining the percentage of reads which map uniquely (Supplemental Fig. S1E). Notably, snCUT&RUN produced better mapping rates after deduplication (15.35% for H3K27ac and 15.62% for H3K4me3) compared with a median of 10.92% and 0.25% with scCUT&Tag and uliCUT&RUN, respectively (Kaya-Okur et al. 2019; Patty and Hainer 2021). The low percentage seen with uliCUT&RUN could be attributed to loss of material with uliCUT&RUN, in which low template concentrations during PCR can cause an increase in artifacts (Ruiz-Villalba et al. 2017).

Finally, we used ChIPseeker (Yu et al. 2015) to examine the genomic annotation of peaks called from the snCUT&RUN, scCUT&Tag, and uliCUT&RUN data sets. As expected, H3K4me3 peaks localized mostly at the promoter region, whereas H3K27ac localized at promoters, introns, and distal intergenic regions, in accordance with known localization of H3K4me3 (active and inactive promoters) and H3K27ac (active promoters and enhancers) (Supplemental Fig. S1F). The localization of peaks follows the trend seen with scCUT&Tag data, whereas uliCUT&RUN had lower localization of H3K4me3 at promoters than expected. Altogether, our benchmarking results show that snCUT&RUN performs similarly or, in many cases, outperforms other available single-cell histone modification profiling methods.

### Copy number amplifications may drive epigenetic reprogramming during metastatic progression in HNSCC

**Figure 2.**
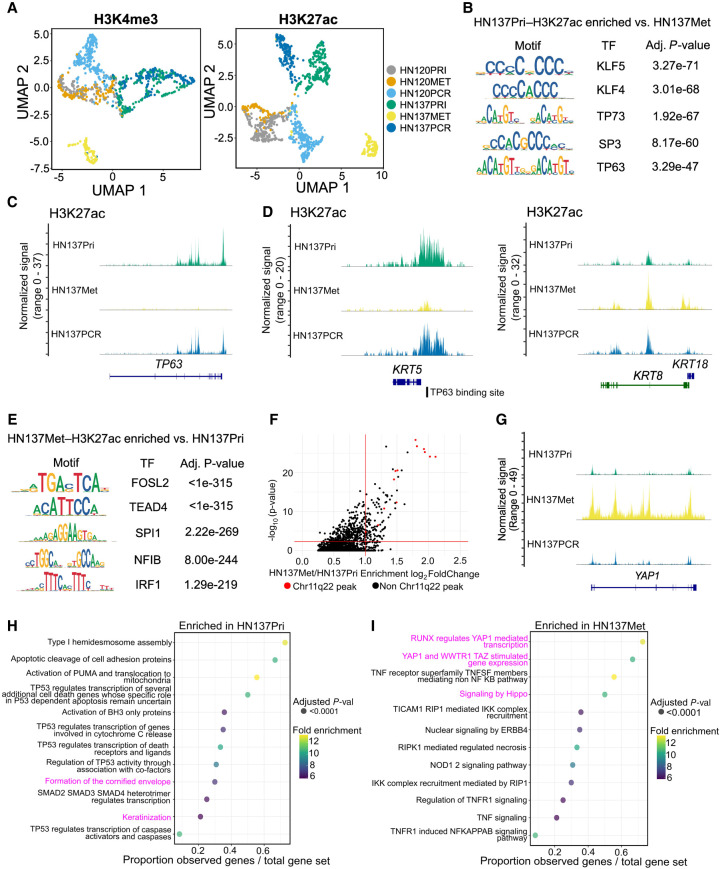
Epigenomic changes during HNSCC progression suggest distinct, patient-specific epigenetic drivers of tumor evolution. (*A*) UMAP embedding for H3K4me3 (*left*) and H3K27ac (*right*) for HN120 and HN137 single-cells. (*B*) Enriched transcription factor (TF) motifs for HN137Pri derived from H3K27ac peaks. (*C*) Coverage plot of H3K27ac signal at the *TP63* locus, showing loss of H3K27ac in HN137Met. (*D*) Coverage plots of H3K27ac signal at the *KRT5* (*left*) and *KRT8/18* (*right*) loci. TP63 binding site near the *KRT5* promoter is indicated. (*E*) Enriched TF motifs for HN137Met. (*F*) Top enriched H3K27ac peaks in HN137Met, with peaks at the Chr11q22 locus containing *YAP1* highlighted in red. (*G*) Coverage plot of H3K27ac signal at the *YAP1* locus. (*H*,*I*) Dot plot indicating the REACTOME pathways enriched in HN137Pri (*H*) and HN137Met (*I*). Highlighted in magenta are enriched terms relating to differentiation in HN137Pri- and YAP1-mediated loss of differentiation in HN137Met.

Having established the technical validity of snCUT&RUN, we utilized the R packages Signac and Seurat (Stuart et al. 2019, 2021; R Core Team 2024) for an integrated analysis of snCUT&RUN data on previously established isogenic patient-derived HNSCC models that represent primary (Pri), metastatic (Met), and cisplatin-resistant primary tumor (PCR) cell lines from two individual patients (HN137 and HN120) (Supplemental Table 1; Chia et al. 2017; Sharma et al. 2018). We used a set of filtering parameters (for details, see Methods) to eliminate low-quality cells. After filtering, single-cell profiles of 1107 and 1048 cells for H3K4me3- and H3K27ac-specific snCUT&RUN data, respectively, were used for downstream analysis. UMAP embedding of H3K4me3 and H3K27ac showed slightly different patterns and varying degrees of overlap between the cell types (Fig. 2A). From H3K4me3 profiles, HN120Pri and HN120Met cells were indistinguishable, and a similar observation was made when comparing HN137Pri and HN137PCR cells (Fig. 2A, left).

In contrast, the PDCs were organized into more distinct clusters when H3K27ac profiles were used for clustering (Fig. 2A, right), suggesting that H3K27ac localization (active promoters and enhancers) is more divergent between primary and progressed cell states compared with promoter activity alone. Notably, HN120PCR and HN137Met seemed to have distinct profiles for both H3K4me3 and H3K27ac, indicating significant differences in their chromatin states compared with other PDCs. We confirmed our clustering results by calculating Pearson correlations between the H3K27ac/H3K4me3 profiles of the different cell types (Supplemental Fig. S2A,B). Furthermore, we did not detect significant batch effects in our clustering (Supplemental Fig. S2C,D).

Next, we sought to infer how changes in epigenetic landscape could drive HNSCC progression using the snCUT&RUN data. After Signac-integrated peak calling, we obtained a set of 606,594 H3K27ac and 391,115 H3K4me3 peaks present in at least one cell line (Supplemental Fig. S3A,B). Out of these peaks, most either are cell line–specific or are shared between all cell lines. However, snCUT&RUN was able to capture peaks that were shared by primary tumor and matched progressed cell lines (Supplemental Fig. S3C,D). Shared peaks among all cell lines located primarily to their expected genomic localization, whereas a small number of sample unique peaks were also observed (Supplemental Fig. 3E,F).

Among the primary tumor/progressed paired cell lines with the starkest H3K27ac/H3K4me3 profile differences are HN137Pri and HN137Met (Fig. 2A). TF motif binding analysis in HN137Pri revealed an enrichment of TP63, a key lineage-determining regulator of epidermal keratinocyte identity (Qu et al. 2018), and KLF4, which was previously shown to regulate differentiation in the basal layer of the oral epithelia (Fig. 2B; Segre et al. 1999). Upon plotting the H3K27ac coverage signal, we observed near loss of H3K27ac at the *TP63* locus, indicating downregulation of *TP63*, and thereby loss of lineage fidelity, in HN137Met (Fig. 2C). Because *TP63* is known as a key regulator of basal keratinocyte identity, we also focused on the keratin (KRT) loci and found a near loss and gain of H3K27ac at the *KRT5* and *KRT8/18* loci, respectively, in HN137Met (Fig. 2D). KRT5/KRT18 immunofluorescence (IF) corroborated our findings from snCUT&RUN data (Supplemental Fig. S4A). Furthermore, we confirmed the downregulation of *TP63* and *KRT5*, as well as the upregulation of *TEAD4* and *KRT8/18* during HNSCC metastasis, upon analyzing two previously published scRNA-seq data sets from primary and metastatic HNSCC (Supplemental Fig. S4B–J; Puram et al. 2017; Sharma et al. 2018). We found an annotated TP63 binding site near the *KRT5* promoter, supporting the notion of *KRT5* expression being driven by *TP63*. Additionally, H3K27ac profiles of the *KRT5* and *KRT8/18* loci in HN120 PDCs aligned with the IF-based protein expression (Supplemental Figs. S4A, S5A). Moreover, we observed concordant H3K4me3 activity at the *KRT5/8/18* loci of HN120 and HN137 PDCs (Supplemental Fig. S5B). Hence, the simultaneous presence (or absence) of H3K4me3 and H3K27ac is a strong indicator of epithelial cell identity. The findings of activating epigenetic marks in the *KRT8/18* locus in HN137Met are significant, because high *KRT8* expression has been associated with detachment of cells from tumors and seeding of lymph node metastasis in HNSCC (Matthias et al. 2008).

The TFs enriched in HN137Met include members of the TEAD family such as *TEAD4*, a downstream activator in the Hippo pathway, and *FOSL2*, a regulator in cellular differentiation (Fig. 2E). Visualization of TF activities at single-cell level showed loss of *TP63* and *TEAD4* in HN137Met and HN137Pri, respectively (Supplemental Fig. S5C). Previously, it was shown that TEAD can repress *TP63* promoter activity and protein expression (Valencia-Sama et al. 2015). We further assessed the differential peaks between HN137Met and HN137Pri. The top enriched peaks in HN137Met were predominantly located at the Chr11q.22 locus containing *YAP1* (Fig. 2F). Increased activity of *YAP1*, in conjunction with its binding partner TEAD, was previously shown to promote proliferation and metastasis in multiple cancers, including HNSCC (Lamar et al. 2012; Chia et al. 2017; Omori et al. 2020). We also found a significant increase in H3K27ac activity of *YAP1* (Fig. 2G). *YAP1* is frequently amplified in HNSCC, and indeed, we have previously reported *YAP1* to be amplified in HN137Met compared with the patient-matched HN137Pri (Chia et al. 2017; Shin and Kim 2020). Next, we assessed top differential enriched peaks (*P* < 0.005) in HN137Pri and HN137Met and used the R package rGREAT (McLean et al. 2010; Gu and Hübschmann 2023) to look for enriched biological pathways. Top enriched REACTOME pathways in HN137Pri related to *TP53* activity (a member of the same family as *TP63*) and keratinocyte differentiation, whereas top pathways in HN137Met include activation of the Hippo pathway, which includes *YAP1* and TEAD, and TNF-mediated activation of NF-kB (Fig. 2H,I). Altogether, these results suggest that H3K4me3/H3K27ac profiles derived from snCUT&RUN could be used to confirm phenotypical changes occurring between primary and progressed HNSCC. Furthermore, the results indicate that considerable changes in epigenetic modifications could be associated with focal copy number amplifications and that a singular focal amplification, such as what was observed with *YAP1*, could lead to epigenetic reprogramming of HNSCC cells to acquire metastatic capabilities. In contrast, HN137PCR cells retained H3K27ac profiles at the *TP63*/*YAP1*/*KRT5*/*KRT8* loci, suggesting a distinct mode of progression not involving *YAP1* amplification.

### Genetic–epigenetic alterations correlate with changes in gene expression

**Figure 3.**
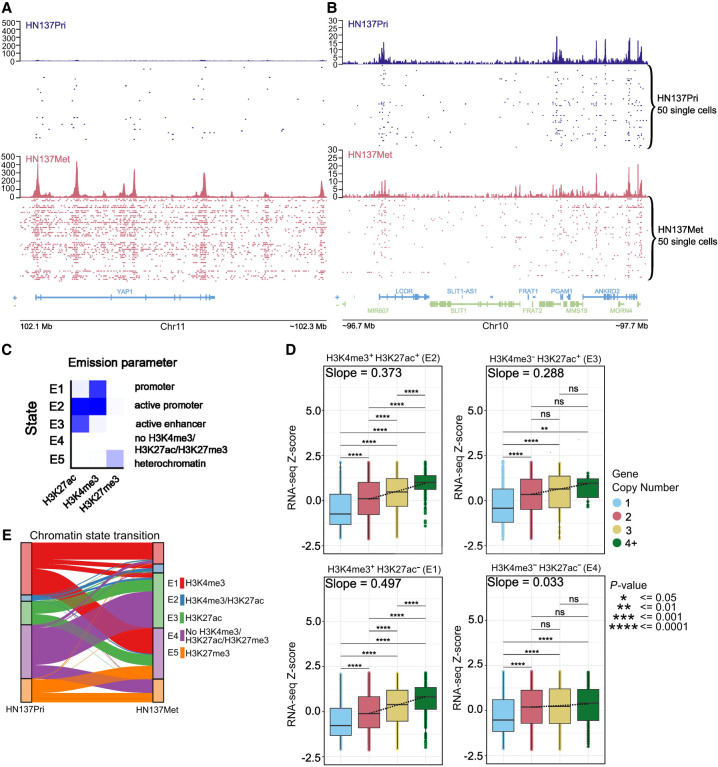
Regulation of gene expression through the interaction between copy number variations and chromatin state. (*A*) Distribution of unique H3K27ac reads in single cells at the *YAP1* locus in HN137Pri (blue) and HN137Met (red). Bulk H3K27ac signal and H3K27ac signal in single cells are shown. (*B*) Distribution of unique H3K27ac reads in a random, non–copy number different locus. (*C*) ChromHMM results identifying five chromatin states consisting of combinations of H3K4me3, H3K27ac, and H3K27me3 modifications in HN120 and HN137 PDCs. (*D*) Boxplots showing HN137Met RNA-seq gene expression *Z*-score values, stratified by chromatin states. The genes were further grouped by copy number (CN) states: CN = 1, heterozygous deletion; CN = 2, normal diploid; CN = 3, low copy number gain; and CN = 4+, copy number amplification. Dashed lines represent quantile regression results of RNA-seq *Z*-scores in between groups with CN = 2, CN = 3, and CN = 4+. Slope values are also indicated. (*E*) Alluvial plot showing chromatin state transition between HN137Pri and HN137Met. Unmodified chromatin (E4; purple), which remained unmodified during metastatic progression, was excluded from the plot for visibility purposes. (**) *P*-value ≤ 0.01, (***) *P*-value ≤ 0.001, (****) *P*-value ≤ 0.0001

Because our data suggested that genetic drivers such as *YAP1* amplification could lead to changes in the epigenome, we further explored how changes in gene copy number (CN) and the combination of active histone marks, such as H3K4me3/H3K27ac, could correlate with alterations in gene expression. We first visualized H3K27ac signal of single cells at the *YAP1* locus for HN137Pri and HN137Met and confirmed our previous findings from Signac in which we observed a marked increase in H3K27ac activity in the *YAP1* signal in HN137Met (Fig. 3A). In contrast, regions without copy number differences do not exhibit significant differences in H3K27ac signal (Fig. 3B). These observations reflect the copy number amplification at the *YAP1* locus in HN137Met. Thus, snCUT&RUN not only can reveal epigenetic changes but also point toward regions potentially affected by focal copy number amplifications. Several other loci with potential focal amplifications, including *EGFR* on Chr 7, were also detected (Supplemental Fig. S6).

Next, to correlate chromatin profiles with gene expression and copy number variations, we performed bulk RNA-seq and whole-exome sequencing (WES) on all PDCs. DNA copy number variation (CNV) has been reported to correlate with gene expression changes. Indeed, we observed a clear positive correlation between the PDC gene expression *Z*-score and the CNs for the same genes (Supplemental Fig. S7A). These results corroborate the notion that changes of gene expression in key cancer genes during HNSCC progression may arise from CNVs. Nevertheless, we hypothesized that epigenetic modifications in such genetically altered loci may further modulate the expression of genes that reside within the same loci. This effect could be compensatory (epigenetic silencing of an amplified gene or epigenetic activation of a deleted gene), or conversely, it could accentuate the effect of CNV (epigenetic activation of the amplified gene or silencing of the remaining copy of a deleted gene). To investigate these hypotheses, we first used ChromHMM (Ernst and Kellis 2012) to perform joint HN120 and HN137 analysis based on pseudobulk H3K4me3, pseudobulk H3K27ac from snCUT&RUN, as well as bulk H3K27me3 CUT&RUN data. ChromHMM results revealed five chromatin states, reflecting distinct chromatin state annotations: weak promoter (E1, H3K4me3 only), active promoter (E2, H3K4me3+/H3K27ac+), active enhancer (E3, H3K27ac only), heterochromatin (E5, H3K27me3 only), and unmodified regions with none of the probed chromatin marks present (E4) (Fig. 3C). Results from separating ChromHMM analysis by individual patient largely follow the results when data from both patients were combined (Supplemental Fig. S7B,C). However, we observed subtle patient-to-patient differences, including the small presence of H3K4me3 at H3K27me3 loci in patient HN137 otherwise not observed in patient HN120 data, possibly indicating that a small number of H3K4me3/H3K27me3 bivalent promoters may be present in these cells (Supplemental Fig. S7C).

We then combined gene CN, chromatin state annotation, and gene expression data to investigate how chromatin state and gene CN may, dependently upon each other, influence gene expression during HNSCC progression. The results showed that only in the presence of activating epigenetic marks did the effects of higher CN on gene expression reach its highest levels (mean *Z*-score difference between genes with CN of 4+ vs. genes with CN of 2 = 0.84 in the H3K4me3^+^/H3K27ac^+^ group and 0.13 in the H3K4me3^−^/H3K27ac^−^ group) (Fig. 3D; Supplemental Fig. S7D). Genes that do not have an activating epigenetic mark have a more muted increase of gene expression *Z*-score with increasing CN. Our results therefore underscore the importance of epigenetic modifications to modulate the effects of CNVs and showed an additive effect of CNVs and epigenetic modifications on gene expression during HNSCC progression. These results further highlight the need to consider both CNVs and epigenetic modifications on gene regulatory elements to understand factors influencing the progression of HNSCC more comprehensively and accurately.

Next, we decided to interrogate how epigenetic profiles at single-cell resolution could assess the function of chromatin state dynamics in driving HNSCC progression from primary cancers to a progressed state (metastatic or treatment resistant). We therefore analyzed genome-wide chromatin state transitions between paired, patient-matched primary tumor, and progressed PDCs (HN120Pri to HN120Met, HN120Pri to HN120PCR [drug resistant], HN137Pri to HN137Met, and HN137Pri to HN137PCR). We found that most of the epigenome (∼95%) remained devoid of H3K27ac, H3K4me3, and H3K27me3 (E4) during the transition from primary tumor to progressed tumor (Supplemental Table 1). However, across the various transitions from primary tumor to progressed HNSCC, proportionally more regions appeared to have gained H3K27ac, whereas a lower proportion of regions with only H3K4me3 was observed (Fig. 3E; Supplemental Fig. S8A). Previous reports have suggested that alterations in cellular metabolism during cancer progression can modulate the levels of cofactors that are substrates of chromatin-modifying enzymes (e.g., acetyl-CoA for histone acetyltransferase), consistent with our observation of increased H3K27ac marks in progressed state. Metabolic reprogramming in cancer cells have been shown to promote EMT by epigenetic activation of EMT-associated genes (Wang et al. 2020).

Finally, we investigated whether significant changes in gene expression during HNSCC progression correlated with changes in chromatin state. We filtered the top downregulated (log_2_ fold change < −0.5, *P* < 0.001) and upregulated genes (log_2_ fold change > 1, *P* < 0.001) during the HN137Pri > HN137Met transition and calculated the enrichment of gene expression change per chromatin state transition. We found that downregulation of gene expression was indeed associated with the loss of H3K4me3/H3K27ac active marks, whereas genes displaying upregulated expression were associated with a gain of either or both H3K4me3/H3K27ac marks (Supplemental Fig. S8B). Altogether, the data suggest that during HNSCC progression, the gain of H3K27ac in key enhancers as a consequence of a global increase in acetylation may serve as the primary driver of changes in gene expression that promote cell state transitions. Furthermore, our data also suggest that alterations in chromatin states occurring between primary and progressed HNSCC are dynamic and heterogeneous, reflecting the diversity of epigenetic changes during tumor progression. These results further corroborated our previous findings that global changes in the epigenome could lead to change in gene expression, highlighting the need for considering chromatin state changes when exploring mechanisms of HNSCC progression. Finally, these results collectively suggest that changes in gene CN and chromatin states may be interconnected in HNSCC and that multimodal integration of genomic and epigenomic data would be essential to generate a more comprehensive understanding of HNSCC progression.

### Adaptive tumor response may underlie HNSCC progression in absence of genetic drivers

Although we observed a genetic–epigenetic interaction in promoting the HN137Pri > HN137Met transition, we did not observe clear genetic drivers during the HN120Pri > HN120Met, HN137Pri > HN137PCR, and HN120Pri > HN120PCR transitions. Therefore, we performed differential motif and REACTOME pathway analyses to investigate these transitions more in depth. During the HN120Pri > HN120Met transition, we observed enriched motifs for several stemness-modulating TFs such as the POU-domain containing proteins POU5F1, POU2F2, and POU2F3, whereas the NOTCH pathway and the P2Y receptor pathways were found to be enriched in HN120Met (Supplemental Fig. S9A,B). These observations are interesting as activation of both pathways has been implicated in the progression of HNSCC and other cancer types (Fukusumi and Califano 2018; Kim et al. 2020; Woods et al. 2020; Zhou et al. 2022). Meanwhile, in the HN137Pri > HN137PCR transition, enriched TF motifs included motifs for the AP1 TF such as JUND, as well as BATF3 and TEAD4 TFs (Supplemental Fig. S9C). Enriched REACTOME pathways included recurrent terms related to the TGFB pathway, such as SMAD signaling and TGFB signaling (Supplemental Fig. S9D). Terms related to HIF1A and tensin (TNS) proteins were also found, and both proteins have been shown to enhance the TGFB pathway activation, leading to increased oncogenic capabilities of HNSCC tumors (Zhao et al. 2024). Separately, it is well known that activation of HIF1A and the TGFB pathway can mediate platinum resistance in multiple cancers (Li et al. 2019; Huang et al. 2021). Activation of the TGFB signaling led to a stemness state and epithelial-to-mesenchymal (EMT) transition, both of which were correlated with cisplatin resistance (Colak and Ten Dijke 2017; Ashrafizadeh et al. 2020). Meanwhile, HIF1A reduces oxidative stress, one of the mechanisms that drives the efficacy of cisplatin in HNSCC (Yu et al. 2020). Similar stemness-related TF motifs were found in the HN120Pri > HN120PCR transition, including *POU2F1* (Supplemental Fig. S9E). POU2F1 deficiency led to higher levels of intracellular reactive oxygen species (ROS) and hypersensitivity to oxidative and genotoxic stress; as earlier mentioned, one of the mechanisms through which platinum compounds like cisplatin induces antitumor effects in HNSCC (Vázquez-Arreguín et al. 2019; Yu et al. 2020). Upregulation of POU2F1 activity was also associated with worse prognosis and oxaliplatin resistance in colon cancer (Lin et al. 2022). Meanwhile, enriched REACTOME pathways included terms related to senescence (Supplemental Fig. S9F). These results point to an adaptive HNSCC response toward cisplatin through dedifferentiation after a transient senescent stage. Earlier, a transient senescent stage was correlated with higher levels of ROS, but conversely, lower ROS levels in stem-like states were observed (Achuthan et al. 2011).

Overall, building on our previous results on the HN137Pri > HN137Met transition, our observations indicated a general role of stemness and dedifferentiation in promoting HNSCC progression. However, in the absence of a clear genetic driver such as was observed in HN137Met, we hypothesize that the tumor can mount an adaptive response through metabolic reprogramming and the selection of cell subpopulations with certain epigenetic states that provides these subpopulations with a fitness advantage to metastasize or resist drug therapy.

### Epigenome profiling at single-nucleus resolution identifies subpopulations of primary HNSCC tumor cells with higher propensity for metastatic progression

Our results above reveal that both genetic changes, exemplified by CNV, and epigenetic flux work in tandem to rewire the gene expression network that drives cancer progression. Amplifications on key oncogenes (e.g., *YAP1*) that associate with crucial TFs could have a disproportionate effect on changing the global epigenetic landscape through interactions with histone writers. However, the results of the HN120Pri > HN120Met, HN137Pri > HN137PCR, and HN120Pri > HN120PCR analysis demonstrated that in the absence of a clear genetic driver, microenvironmental pressures may lead to primary tumor cancer cell adaptation by acquiring a range of epigenetic states, and this cellular plasticity could confer survival and growth advantage under the selective pressure of metastasis or drug treatment. In the context of HNSCC progression, some cell subpopulations, especially those at the invasive borders of primary tumors, have been shown to express a partial epithelial-to-mesenchymal (pEMT) transcription program (Puram et al. 2017). Compared with irreversible genetic changes, nongenetic changes could be much more plastic and heterogeneous among cancer cells. We therefore sought to utilize the snCUT&RUN data to explore intratumor epigenetic heterogeneity (ITeH) as a factor driving HNSCC progression in the absence of a clear genetic driver. We first analyzed the transition between HN137Pri and HN137PCR, because these PDCs were found to have higher similarity in H3K4me3/H3K27ac profiles as well as the phenotype (Supplemental Figs. S2A, S4A).

**Figure 4.**
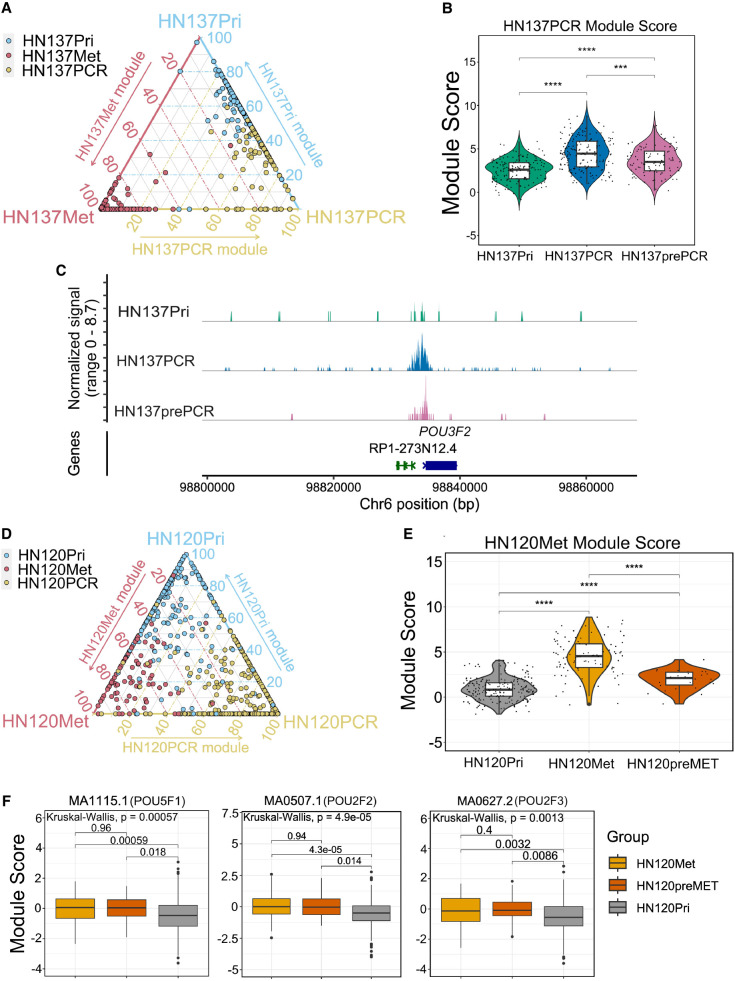
Epigenetic heterogeneity–driven HNSCC progression. (*A*) Ternary plot showing the ratios of Pri/Met/PCR module scores for patient HN137. (*B*) Violin plot depicting the recalculated HN137PCR module score after identification of the HN137prePCR subpopulation. (*C*) CoveragePlot of the *POU3F2* locus showing higher H3K27ac localization in HN137prePCR compared with the remaining HN137Pri cells. (*D*) Ternary plot showing the ratios of Pri/Met/PCR module scores for patient HN120. (*E*) Violin plot depicting the recalculated HN120Met module score after identification of the HN120preMET subpopulation. (*F*) Boxplots of ChromVAR scores of several POU-domain containing proteins enriched in HN120Met, showing that the HN120preMET population has higher activity of these TFs compared with the remaining HN120Pri cells. (***) *P*-value ≤ 0.001, (****) *P*-value ≤ 0.0001.

Analyzing H3K27ac profiles, we found a lower number of differentially enriched peaks (*P*-value < 0.005) in HN137PCR versus HN137Pri (2245), compared with HN137Met versus HN137Pri (4797). Hence, changes in H3K27ac expression during the cisplatin resistance of HN137Pri appeared to be less widespread compared with the metastatic progression of patient HN137. These results point to a gradual change in the epigenome caused by ITeH rather than a genetically driven change during progression to a drug-resistant state. To further investigate ITeH in the cisplatin-resistance progression of HN137Pri, we defined PDC-specific “modules,” which are features consisting of the top 50 differential peaks defining a specific PDC-state. This analysis was inspired by scRNA-seq analysis, in which modules are defined as groups of genes that are part of the same module/program, typically referring to cell type–specific gene expression signatures. After defining H3K27ac modules for each PDC, we then computed ChromVAR deviation *Z*-scores of each module for each individual cell (Schep et al. 2017). In our case, the *Z*-score represents how far the H3K27ac profile of that cell deviates from the average H3K27ac profile of every cell within the peaks as defined by the PDC-specific modules. Analyzing the ChromVAR deviation *Z*-scores of the HN137 isogenic cell lines, we found that each PDC has the highest module score of their individual module (e.g., HN137Met has the highest *Z*-score for the HN137Met module) (Supplemental Fig. S10A). The deviation *Z*-score profiles confirmed that each module is capable of distinguishing identities of individual PDCs. However, we also observed a degree of variability in *Z*-scores within a given cell type, prompting the question whether this represents the possibility of an epigenetically heterogeneous state within the PDCs. We therefore normalized the primary tumor, metastatic, and primary tumor cisplatin-resistant module scores of each single cell and plotted the normalized scores using ternary plots, making use of the three-variable nature of our data to visualize the ratio of Pri/Met/PCR module score per single cell (Fig. 4A). Ternary plot visualization confirmed our previous findings, showing that HN137Pri and HN137PCR cells are in a continual axis based on their module scores. Such continuity was not observed between HN137Pri and HN137Met, suggesting that the HN137Pri → HN137PCR transition is characterized by intrinsic epigenetic intra-tumoral heterogeneity and is not defined by strong genetic/epigenetic drivers, as is the case with HN137Pri → HN137Met progression.

To further investigate epigenetic heterogeneity, we set apart HN137Pri cells with Pri/Met/PCR module score ratio of >0.4 Pri and >0.4 PCR and annotated these cells “HN137prePCR” cells. We obtained 93 HN137Pri cells (representing 37% of the primary population) fulfilling this criterion. HN137prePCR cells were found to have a higher average score of the HN137PCR module compared with the remaining HN137Pri cells, yet still below the average of HN137PCR cells (Fig. 4B). HN137prePCR cells also exhibited H3K4me3 profiles more similar to HN137PCR cells compared with HN137Pri cells at several loci, including *POU3F2* (Fig. 4C; Supplemental Fig. S10B). Altogether, these results could potentially indicate that HN137prePCR cells represent a primary tumor cell subpopulation, which is predisposed toward becoming cisplatin resistant because of their “cisplatin-resistant-primed” epigenetic state.

To exclude UMR count and FRiP as confounding factors of the module scores, we compared the UMR count and FRiP of HN137prePCR cells against the remaining HN137Pri cells, as well as HN137PCR cells. HN137prePCR cells did not have higher UMR count on average compared with the remaining HN137Pri as well as HN137PCR cells (Supplemental Fig. S10C). Furthermore, HN137prePCR cells did not have higher FRiP values compared with HN137PCR and HN137Pri cells (Supplemental Fig. S10D). Hence, UMR count and FRiP were not deemed to be significant factors in determining module scores.

Finally, we investigated whether ITeH could drive the lymph node metastatic progression of patient HN120. In a similar approach to investigate whether ITeH drives the progression of cisplatin resistance in patient HN137, we isolated HN120Pri cells with a >0.4 HN120Pri H3K27ac module ratio and a >0.4 HN120Met module ratio. We observed 26 cells that met this criterion (∼9% of the total HN120Pri population) and relabeled these HN120Pri cells as the “HN120preMET” subpopulation (Fig. 4D). Similar to HN137prePCR, recalculation of the HN120Met module score showed that the HN120preMET cells have a higher average deviation *Z*-score for the HN120Met module compared with the remaining HN120Pri cells, but still lower than HN120Met cells (Fig. 4E). HN120preMET cells exhibited higher TF activity for several of the TFs enriched in HN120Met compared with the remaining HN120Pri cells, including key stemness modulating TFs such as *POU5F1* (also known as *OCT4*) (Fig. 4F; Supplemental Fig. S10E). Overall, the HN120preMET and HN137prePCR analyses support the existence of H3K27ac landscape heterogeneity in HNSCC tumors and suggest that in the absence of strong genetic drivers, tumor adaptation could drive ITeH in primary HNSCC tumors, leading to certain selected, epigenetically primed, subpopulations acquiring a phenotype with higher propensity to progress into a more malignant state.

## Discussion

Intra-tumor heterogeneity of chromatin states can serve as an epigenetic driver of HNSCC progression. However, studies using single-cell methods to profile epigenetic heterogeneity in HNSCC models of cancer progression remain lacking. Here we developed snCUT&RUN, a robust method to profile histone modifications at the single-cell level. We showed that snCUT&RUN could provide insights in chromatin state transitions between isogenic primary and progressed HNSCC PDCs at the level of both global and single-cell resolution. At the global level, we found that generally H3K27ac signal is better capable of distinguishing different PDC cell states, suggesting that active enhancer and promoter landscapes are more unique to each individual cell state compared with just promoter activity alone. Furthermore, our results indicate that the gain of H3K27ac at promoters and/or poised enhancers may serve as critical factors driving tumor progression. We validated that H3K27ac/H3K4me3 activity at the *KRT5* and *KRT8*/*18* loci served as an accurate indicator of epithelial identity and that snCUT&RUN can be used to comprehensively analyze a mixture of samples to infer epithelial changes and the accompanying TF motif changes during tumor progression. We showed that these changes are possibly associated with a focal copy number amplification of *YAP1*, which in turn could lead to altered TEAD activity and subsequent epigenetic rewiring. Indeed, an increasing body of evidence shows that genetic and epigenetic factors are closely associated with driving tumor heterogeneity and progression. An example of this interplay between the genome and the epigenome was highlighted in recent studies on extrachromosomal circular DNA (ecDNA) amplifications that amplify not only oncogenes but also functional elements such as enhancers, thereby increasing chromatin accessibility and downstream expression of key oncogenes (Morton et al. 2019; Wu et al. 2019). We further showed that gene expression is affected by both gene CN as well as gene chromatin state, in which the highest gene expression was observed in genes that have higher CN and have active chromatin marks. These results emphasize the complexities of factors that promote tumor progression and highlight the importance of considering both genetic and epigenetic factors that drive cell state plasticity.

However, we noted that some epigenetic changes were more subtle and did not involve wide-scale epigenetic rewiring driven by alterations in the genome. Comparing both HN120Pri and HN120Met as well as HN137Pri and HN137PCR, we did not observe clear lineage infidelity from both the H3K4me3 and H3K27ac profiles. This led to the hypothesis that in some tumors, microenvironmental pressures such as metabolic dysregulation or challenges from the immune system may lead to primary tumor subpopulations to activate certain TF programs to adapt to these selective pressures. This adaptation process inherently leads to heterogeneous chromatin states in the primary tumor, which may confer selective fitness to the subpopulations with an intermediate malignant chromatin state. Indeed, a previous study described a subpopulation of malignant HNSCC cells being in a state of pseudo-epithelial-to-mesenchymal transition (pseudo-EMT), which correlated with cancer progression (Puram et al. 2017). We used the module score analysis and showed that H3K27ac profiles could be used to find such subpopulations, which may have a higher propensity to progress to malignant cell states.

We note that the samples we have used for this study are long-term cell cultures that may have acquired a degree of homogeneity through the course of several passages. However, some evidence suggests that heterogeneity is maintained even in long-term cultured cell lines. For example, our cell line models exhibit chromosomal instability (CIN). CIN is known to lead to heterogeneity in gene expression while at the same time being maintained in long-term cell cultures. In a study by Minussi et al. (2021), the authors performed single-cell clonal outgrowth of the CIN-affected MDA-MB-231 breast cancer cell line and found that karyotype diversity was regained after only 19 passages, suggesting that in vitro cancer cell cultures rediversify their genomes and maintain genetic heterogeneity throughout passaging. Epigenomic heterogeneity was also shown to be maintained in cell lines. Litzenburger et al. (2017) observed through scATAC-seq experiments that the leukemic cell line K562 exhibited epigenomic heterogeneity, which has functional consequences such as drug response. Hence, it is likely that our cell line models exhibit sufficient intra-tumor heterogeneity to make single-cell assessments useful.

**Figure 5.**
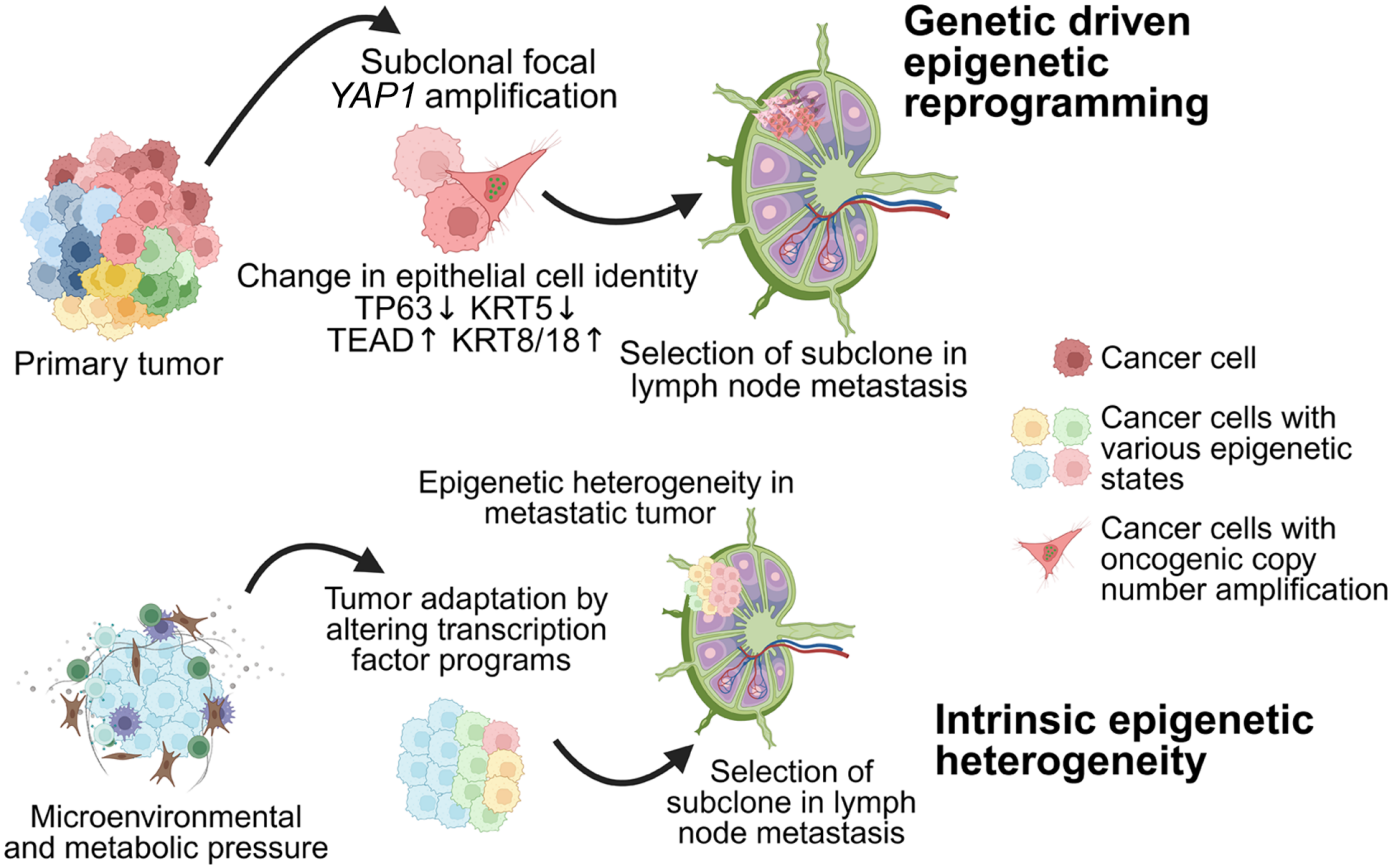
Model of diverse ways in which epigenetic changes can lead to adaptation of cellular phenotype to acquire a more aggressive state and promote HNSCC progression. Created with bioRender (https://www.biorender.com/) under publication license GS26ET99NE.

Altogether, based on the observations from snCUT&RUN analysis on isogenic primary and progressed cell states in HNSCC, we propose the following working model for epigenetic control of HNSCC progression (Fig. 5). First, in the presence of a strong genetic driver, for example, copy number amplification, epigenetic reprogramming can occur, which may result in the selection of cells with favorable gene expression signatures to progress. Second, in the absence of strong genetic drivers, microenvironmental pressures may drive a primary tumor adaptation response, leading to intra-tumor epigenetic heterogeneity. ITeH results in the emergence of transitionary subpopulations that are more prone to progress into a more malignant or resistant cell state. Future characterization of such subpopulations can result in the identification of prognostic biomarkers and therapeutic targets that can be used for better patient stratification and the development of novel intervention strategies to block progression to malignant states.

At present, snCUT&RUN is positioned as a medium-throughput assay. Slightly more than 2000 FACS-sorted single cells were profiled in this work. Although it may not achieve the throughput of scCUT&Tag assays, its high sensitivity is a major advantage for uncovering subtle cell-to-cell epigenetic heterogeneity, such as those found within a tumor. We envisage that future improvements, such as automation of single-cell library preparation with an automated liquid handler, would further enable snCUT&RUN assay to be adopted by the wider scientific community.

## Methods

### Cell materials and tissue culture

HN120Pri, HN120Met, HN137Pri, and HN137Met patient-derived cells (PDCs) used for this study were retrieved from a biobank previously generated using described methods (Chia et al. 2017). Likewise, HN120PCR and HN137PCR cells were generated as part of an earlier study (Sharma et al. 2018). The identities of the primary cell lines and their derivatives were confirmed using short-tandem repeat (STR) profiling (Supplemental Table 2). Normal tissue samples for these patients were not available for experiments. Cells were cultured with RPMI medium supplied by 10% fetal bovine serum (FBS) and 1% penicillin/streptomycin. Medium was replaced every 2–3 days. Cells were cultured in 37°C and 5% CO_2_ and passaged when cultures reached ∼90% confluency. Cells were tested for mycoplasma and were only used for experiments after being confirmed to be mycoplasma negative.

### Immunofluorescence

Cells were cultured in 96-well plates with 10,000–20,000 seeding density for 48 h. Fixation was done in acetomethanol 1:1 ratio for 10 min at −20°C. After washing 3× with 1× PBS, blocking was done with 2% BSA/0.1% Triton X-100/PBS for 1 h+. The following primary antibodies were used: ab17130 (KRT5, 1:100 dilution) and ab32118 (KRT18, 1:100 dilution). Cells were incubated overnight at 4°C. Alexa Fluor488/594 and Hoechst33342 were used during secondary antibody incubation for 30 min at 37°C. Cells were imaged with the Nikon EclipseTi inverted microscope using widefield setting and 20× magnification.

### Single-nucleus CUT&RUN

Cells cultured to ∼80% confluence in T75 flasks were harvested and washed 2× in sucrose buffer (5% sucrose, 1% BSA, 20 mM HEPES at pH 7.5, 150 mM sodium chloride, 0.5 mM spermidine, 1× cOmplete, Mini, EDTA-free protease inhibitor cocktail; Roche 04693159001) at 200*g* for 3 min. For each antibody tested, 1.5 × 10^6^ cells were dispensed into Protein LoBind tubes (Eppendorf 0030108442), pelleted, and suspended in 1× ice-cold lysis buffer (sucrose buffer containing 0.005% NP-40, 0.005% digitonin, 2 mM EDTA, and 10 mM sodium butyrate). The amount of cell lysis was detected using the trypan blue exclusion assay, and remaining intact cells were lysed by titrating-in 2× cold lysis buffer (sucrose buffer containing 0.01% NP-40, 0.01% digitonin, 2 mM EDTA, and 10 mM sodium butyrate). Nuclei were pelleted then suspended in sucrose buffer containing 1:100 antihistone H3 (trimethyl K4) antibody (abcam ab213224) or antiacetyl-histone H3 (Lys27; Merck MABE647) and incubated ≥1.5 h at 4°C with intermittent agitation. Nuclei were washed 2× with sucrose buffer then suspended in 700 ng/ml pA-MN (a kind gift from Steven Henikoff) in sucrose buffer and incubated for 60 min at 4°C. Pelleted nuclei were suspended in nuclear stain buffer (sucrose buffer containing 1:4000 Alexa Fluor 647 antinuclear pore complex proteins antibody [BioLegend 682204] plus 4 µM ethidium homodimer [Invitrogen E1169]) and incubated for 10 min at room temperature (RT). After 2× washes with sucrose buffer, the nuclei were suspended in low-salt buffer (20 mM HEPES at pH 7.5, 0.5 mM spermidine). FACS was performed with an MoFlo Astrios cell sorter (Beckman Coulter) operated using Summit software. Single nuclei were gated first using forward and side scatter pulse area parameters (FSC-A and SSC-A), aggregates were excluded using pulse width (FSC-W and SSC-W), and then isolated nuclei were gated based on AF647 and ethidium homodimer fluorescence. Nuclei were sorted directly into 3 µL of calcium buffer (10 mM calcium chloride, 3.5 mM HEPES at pH 7.5) in PCR strip tubes for DNA digestion. As a QC measure, nuclei were also sorted into buffer on flat-well optical plates (4titude 4ti-0970/RA) to check for wells with more than one isolated nucleus via fluorescence microscopy. For each array of 96 PCR tubes, positive and negative control tubes were included composed of 1000 dispensed nuclei or buffer-only wells, respectively. To stop digestion, 1 µL of 4× STOP buffer (600 mM sodium chloride, 80 mM EGTA, and 0.05% digitonin) was added to the sides of the strip tubes; the tubes were pulse-centrifuged, mixed by touching the sides of the tubes against a rotating vortex, and then pulse centrifuged a second time. Tubes were then incubated for 30 min at 37°C.

Library prep on single nuclei was performed as follows: 0.8 µL 1× end repair and A-tailing buffer/enzyme mix (KAPA hyperprep kit, 07962363001) was added to the sides of the tubes, mixed as before, and then incubated for 15 min at 12°C, 15 min at 37°C, for 90 min at 58°C, and then 8°C on hold. Adapter ligation was performed by adding 0.5 µL 300 nM unique dual indexed adapter (NEXTFLEX NOVA-514150 or NOVA-514151) and 3.6 µL 1× ligation buffer/enzyme mix (KAPA hyperprep kit, 07962363001) to the sides of the tubes, mixed as before, and then incubating for 16 h at 4°C.

To remove excess adapters, the volumes were adjusted to 20 µL with 10 mM Tris (pH 8.0); 20 µL 2× SPRI beads (MagBio Genomics AC-60050; 2× SPRI beads: 1 volume SPRI beads magnetically separated, one-half volume PEG/NaCl solution removed [and stored], and then beads resuspended, thus increasing the surface area of beads for DNA to adsorb to) were added to inverted fresh strip tube caps; the caps were then affixed; and the tubes mixed as before then incubated at RT for 2 h. SPRI beads were separated by tethering the strip tubes to N50 grade neodymium magnets affixed to a ferrous metal rig for stability. The supernatant was removed; the beads were washed twice with 80% ethanol and then allowed to dry for 3 min at RT. The tubes were removed from the magnets and beads suspended in 20 µL 10 mM Tris (pH 8.0). Twenty-two microliters of PEG/NaCl was added, mixed into the beads with pipetting, and then incubated for 2 h at RT (or tubes left overnight at 4°C, brought back to RT, and then incubated for 2 h at RT). Beads were washed twice with 80% ethanol, air-dried for 3 min and then suspended in 7.5 µL 10 mM Tris (pH 8.0), and DNA allowed to elute for ≥10 min at RT. Hereafter, the positive control tubes were treated separately and not pooled with single nuclei libraries and no template controls. The beads were magnetically separated as above and pooled eluate added to PCR strip tubes containing 2× KAPA HiFi HotStart ReadyMix (Roche 07958935001) and 2 mM each of P5 (AATGATACGGCGACCACCGAGATCTACA*C) and P7 (CAAGCAGAAGACGGCATACGAGA*T) primers with phosphorothioate bond as indicated with asterisk. PCR was performed with a thermocycler using a heated lid under the following cycling conditions: 45 sec at 98°C; 19 cycles of 15 sec at 98°C and 10 sec at 60°C; 1 min at 72°C; and 8°C on hold.

Pooled library DNA was concentrated as follows: amplified DNA was pooled together; 400 µL aliquots were dispensed into 1.5 mL tubes; 400 µL phenol:chloroform:isoamyl alcohol 25:24:1 was added; and the samples were vortexed. The mixture was then transferred to MaXtract high-density tubes (Qiagen 129056) and centrifuged for 5 min at 16,000*g* at RT. Four hundred microliters of chloroform was then added to the MaXtract tubes, mixed by inverting several times, and then centrifuged for 5 min at 16,000*g* at RT. The aqueous phase from each MaXtract tube (∼400 µL) was then transferred to 1.5 mL tubes containing 2 µL of 2 µg/µL glycogen (Roche 10901393001), 1 mL ethanol was added, the tubes vortexed, then incubated at −20°C overnight. DNA was pelleted by centrifuging at 20,000*g* for 10 min at 4°C, washed with 1 mL 100% ethanol, and then air dried for at least 15 min at RT. Pellets were suspended and combined in a total volume of 100 µL 10 mM Tris (pH 8.0). Adapter dimers and excess primers were removed by adding 90 µL SPRI beads and incubating for 15 min at RT, magnetically separating the beads, washing twice with 80% ethanol, and eluting with 50 µL 10 mM Tris (pH 8.0). DNA was allowed to rebind to the beads by adding 45 µL PEG/NaCl; the beads were incubated for 15 min at RT, magnetically separated, washed twice with 80% ethanol, and then eluted in 50 µL 10 mM Tris (pH 8.0). A third SPRI bead purification with a ratio of 1:1 was found to be necessary to remove residual adapter dimers prior to sequencing (i.e., 50 µL PEG/NaCl added to the DNA/bead mixture and two ethanol washes performed). Finally, the DNA was eluted in 20 µL 10 mM Tris (pH 8.0). All libraries were sequenced using paired-end sequencing on an Illumina MiSeq, with samples processed using a MiSeq reagent kit V3 (150 cycles; Illumina MS-102-3001).

### Bulk cell CUT&RUN

Bulk cell CUT&RUN was performed in the same manner as snCUT&RUN with the following alterations. After incubating with pA-MN, cells were washed twice in BSB followed by one wash in low-salt buffer. After pelleting, most of the supernatant was removed, leaving a small volume, and the pellet was suspended to a slurry by gentle agitation. Thirty-seven microliters of calcium buffer was added to activate pA-MN and the samples incubated in a prechilled heat-block in ice water for 30 min. The reaction was stopped by adding 12.5 µL 4× STOP buffer and nucleosomes allowed to diffuse for 30 min at 37°C. Fifty microliters was removed to a PCR tube containing 10 µL 1× end repair and A-tailing buffer/enzyme mix (KAPA hyperprep kit 07962363001) and incubated for 15 min at 12°C, 15 min at 37°C, 90 min at 58°C, followed by 8°C on hold. Five microliters of 15 mM TruSeq DNA single indexes (SKU 20015960) and 45µL 1× KAPA hyperprep enzyme/buffer mix were added to each sample and incubated for 16 h at 4°C. Two microliters of 10% SDS plus 2 µL Proteinase K (Thermo Fisher Scientific EO0492) were added and incubated for 60 min 37°C. Excess adapters and adapter dimers were removed via two successive washes with SPRI beads. One hundred ten microliters of SPRI beads (MagBio Genomics AC-60050) were added to each sample and incubated for 15 min at RT. Beads were magnetically separated and washed twice with 80% ethanol. After air drying for 5 min, DNA was eluted from the beads by suspended in 50 µL 10 mM Tris (pH 8.0). DNA was rebound to the beads by adding 60 µL 20% PEG 8000, 2.5 M NaCl and incubating for 15 min. Beads were separated and washed as before and then suspended in 20 µL 10 mM Tris (pH 8.0). To each purified library, 25 µL 2× KAPA HiFi HotStart ReadyMix was added plus 5 µL TruSeq single-index PCR primer cocktail. PCR was performed using the following cycling conditions: 45 sec at 98°C; 12 cycles of 15 sec at 98°C and 10 sec at 60°C; 1 min at 72°C; and 8°C on hold. Bulk cell libraries were purified using two successive rounds of SPRI bead purification as above, using 1:1.1 and then 1:1.2 ratios of sample to beads, and eluting with 20 µL 10 mM Tris (pH 8.0).

### snCUT&RUN data preprocessing and QC measurements

Raw FASTQs of single cells were mapped to the human GRCh38 (hg38) reference genome with Bowtie 2 (Langmead and Salzberg 2012, 2) v2.3.5.1 and the following settings: ‐‐end-to-end ‐‐very-sensitive ‐‐no-mixed ‐‐no-discordant -q ‐‐phred33 -I 10 -X 700. The MarkDuplicates tool from GATK v4.1.4.1 (McKenna et al. 2010) was used to mark and remove duplicate reads, and SAMtools v1.10 (Li et al. 2009) was used to index the BAM files. BEDTools v2.27.1 (Quinlan and Hall 2010) was used to convert BAM files to BED and bedGraph. The unique number of UMRs was calculated by first converting the deduplicated single-cell BAM file to a BED file, which in turn was used to create a TagAlign file. The number of paired-end reads in the TagAlign file was then counted as the number of UMRs. To calculate the fraction reads in peaks, we first merged the single-cell data into pseudobulk and then normalized based on the sample with the lowest read number. We then called peaks using MACS2 (Zhang et al. 2008) with the following settings: BAMPE ‐‐nomodel -B -p 0.05 ‐‐min-length 500 ‐‐max-gap 400 ‐‐SPMR ‐‐call-summits. The fraction of reads that overlap the called peaks was then divided by the total number of reads to calculate the FRiP. Equally, we calculated the fraction of peaks in blacklisted genomic regions (Amemiya et al. 2019). For comparing snCUT&RUN with currently available methods, previously published data were accessed from the NCBI Gene Expression Omnibus (GEO; https://www.ncbi.nlm.nih.gov/geo/) under accession numbers GSE124557 (scCUT&Tag), GSE158435 (CoTECH), GSE224560 (Droplet Paired-Tag), and GSE111121 (uliCUT&RUN).

### Signac/Seurat analysis

To create Signac fragment files, individual single-cell BAM files were first processed into BED files with the following columns: chr, start, end, cell barcode, and a fifth column with the number 1, representing that each row combination only occurred once. Individual BED files were concatenated and sorted with BEDTools and indexed with tabix. Data from H3K4me3 and H3K27ac were processed separately. The fragment file was then converted to a sparse bin matrix with Signac's GenomeBinMatrix() function in R4.0, specifying a bin size of 10kb (R Core Team 2024). Subsequently, the bin matrix was used to create a chromatin assay in Signac. Cells were excluded from analysis with the following criteria: UMRs < 1000, UMRs > 100,000, FRiP < 0.25, fraction reads in blacklist > 0. 01, nucleosome signal > 5, and TSS enrichment score < 0.5. Signac standard analysis was performed downstream. Briefly, the matrix was normalized with term frequency inverse document frequency (TF-IDF), and singular value decomposition (SVD) was applied. UMAP embedding was done with the following settings: umap.method = “uwot,” n.neighbors = 10, metric = “Manhattan,” n.epochs = 500, min.dist = 0.1, spread = 1, set.op.mix.ratio = 1, reduction = “lsi,” dims = 2:30. MACS2 peaks were called with the CallPeaks() function, specifying the broad and combine.peaks options set to TRUE, BAMPE ‐‐nomodel -B -p 0.05 ‐‐min-length 500 ‐‐max-gap 400 ‐‐SPMR ‐‐call-summits. Peak calls were visualized with the CoveragePlot() function. To perform motif analysis, first a PFMatrix object was retrieved from the JASPAR2020 package (Fornes et al. 2020). A peak matrix was then created using fragment files and peak calls. Subsequently, the AddMotifs() function was used to construct a motif object containing motif information. ChromVAR deviation *Z*-scores were calculated with the RunChromVAR function. Reactome pathway analysis of peaks was done with the rGREAT package v1.24.0 (McLean et al. 2010; Gu and Hübschmann 2023). For this analysis, only peaks with differential *P*-value of <0.005 were included.

### Bulk RNA-seq

Total RNA was extracted with the Qiagen RNAeasy plus mini kit (74136) and quantified with NanoDrop. Triplicates for each cell line (using different passage numbers) were used. RNA-seq library preparation and directional mRNA-sequencing were carried out by NovogeneAIT. An adapted analytical workflow from Love et al. (2019) was used for the analysis. Briefly, transcripts were quantified using Salmon (Patro et al. 2017) with hg38 genome and GENCODE version 38 as references and default parameters. Quantified transcripts were imported to R with tximeta (Love et al. 2020), and differential analysis was performed with DESeq2 (Love et al. 2014), filtering out genes with fewer than 10 supporting reads. *Z*-scores were calculated with the scale() function after variance normalization with variance stabilizing transformation (VST) to account for count variations caused by highly expressed or lowly expressed genes. The resulting output is a matrix in which rows represent genes, columns represent samples, and values represent *Z*-score. The mean *Z*-score of the three replicates was calculated as the representative *Z*-score for each individual PDC.

### Whole-exome sequencing

WES for HN120Pri, HN120Met, HN137Pri, and HN137Met were done through Macrogen, whereas WES for HN120PCR and HN137PCR was done through NovogeneAIT. In this case, the Agilent SureSelect V6 58 Mb kit was used for library preparation, and samples were sequenced on an Illumina NovaSeq PE150 platform at 12 Gb data/100× exome coverage. Raw WES data were processed through GATK best-practice pipeline, and copy number segmentation and calling were done with CNVKit after normalizing read number to the lowest sample (HN137Met) (Talevich et al. 2016).

### Correlating gene CN, chromatin state, and expression

Single-cell BAM were first aggregated into pseudobulk with SAMtools merge and processed with sorting and indexing. Next, to account for differences in signal arising owing to differences in read number, we normalized the BAM files by downsampling reads to match the read number of the sample with the smallest read number. Normalized BAM files of pseudobulk aggregate H3K4me3 and H3K27ac, as well as bulk H3K27me3 data, were used as input for ChromHMM v1.23 (Ernst and Kellis 2012), using hg38. ChromHMM is a multivariate hidden Markov model–based technique that can model the presence of multiple chromatin marks in the same region of the genome. It first uses the Baum–Welch algorithm to find “hidden chromatin states,” each state reflecting presence or absence of single or multiple histone marks. It then uses the forward–backward algorithm to calculate the posterior probability of a certain genomic region being in a particular chromatin state. Five states were deemed to be the optimal number of states after repeated analysis with multiple numbers of states. Output segment files were binned to 200 bp windows with BEDTools makewindows to allow for comparable analysis across multiple cell lines. To correlate ChromHMM state transitions and gene expression, ChromHMM 200 bp genomic bin outputs were filtered to include only promoter regions (region of 2000 bp upstream of TSS to 3000 bp downstream from TSS) and correlated to the nearest gene with BEDTools closest. The resulting output is a BED-like file with the columns chromosome, bin start, bin end, chromatin state, and gene. Gene symbols were used as identifiers to connect gene chromatin state, gene CN, and gene expression. The *Z*-score matrix and CNVKit .call.cns output were used for the gene expression and gene CN data, respectively. Genes with missing data (e.g., no gene expression or CNV data) were removed from analysis. To assign chromatin state from the 200 bp ChromHMM output, we considered a gene to have an activating mark if more than two bins (400 bp) have H3K4me3 signal, H3K27ac signal, or both. Genes were categorized as having activity of both marks if the modal chromatin state across all bins are H3K4me3^+^/H3K27ac^+^ or when there are similar proportion of H3K4me3^+^/H3K27ac^−^ and H3K4me3^−^/H3K27ac^+^ bins (e.g., 40% H3K4me3^+^/H3K27ac^−^ and 60% H3K4me3^−^/H3K27ac^+^ or vice versa). A gene is categorized as having a single mark if >60% of the bins were annotated as such by ChromHMM. For chromatin state transition analysis, bins that were unmodified in the primary state and that remained unmodified during progression were excluded from analysis. Alluvial plots were plotted with the R package ggalluvial (Brunson 2020). Barplots and boxplots were visualized with the R package ggplot2.

### HN120preMet and HN137prePCR analysis

Differential marker peaks for each individual PDC were found through the FindAllMarkers() function of Seurat. The top-50 peaks were included as features for the “module” unique for each PDC. Subsequently, module scores were back-calculated for each single cell with the AddChromatinModule() function. To plot the ternary plots, first individual module profiles of HN120 and HN137 PDCs were separated. Next, normalized module score profiles of each individual cell were added up to one with the following formula:

$$S_{x\_norm\;}=\;\frac{S_{x}\;}{S_{PRI\_x}+S_{MET\_x}+S_{PCR\_x}},$$

whereby *S*_*x*_ is the raw PRI or MET or PCR module score of that cell, and *S*_(*x_norm*)_ is the normalized PRI or MET or PCR score for that cell. If there were negative values, the absolute value of the lowest negative value was added to the PRI/MET/PCR scores. The package ggtern (Hamilton and Ferry 2018) was then used to visualize the normalized module scores. For HN120Pri, cells were categorized as preMet if cells have a PRI/MET/PCR-normalized module score ratio of >0.4 Pri and >0.4 Met. For HN137Pri, cells were relabeled as HN137prePCR if cells have a PRI/MET/PCR-normalized module score ratio of >0.4 Pri/<0.4 PCR. The ggplot package and the geom_smooth() function were used to calculate the linear regression between HN120Pri and HN120Met module scores with UMR and FRiP.

### Statistical analysis

All statistical analysis was done with R4.0 (R Core Team 2024). The two-sided Wilcoxon test from the stat_compare_means function of the ggpubr package was used to calculate statistical significance of either module scores, UMRs, or FRiP between different PDCCs. A linear regression model was used to analyze the statistical relationship between HN120Met module score and FRiP and UMR. *P*-values are indicated: (*) *P* ≤ 0.05, (**) *P* ≤ 0.01, (***) *P* ≤ 0.001, and (****) *P* ≤ 0.0001.

## Data access

All raw and processed sequencing data generated in this study have been submitted to the NCBI Gene Expression Omnibus (GEO; https://www.ncbi.nlm.nih.gov/geo/) under accession number GSE212250. The code used in this study is available at GitHub (https://github.com/dmuliaditan/sncutnrun) and as Supplemental Code.

## Supplemental Material

Supplement 1

Supplement 2

Supplement 3

Supplement 4

## References

[GR279105WOMC1] Achuthan S, Santhoshkumar TR, Prabhakar J, Nair SA, Pillai MR. 2011. Drug-induced senescence generates chemoresistant stemlike cells with low reactive oxygen species. J Biol Chem 286: 37813–37829. 10.1074/jbc.M110.200675 21878644 PMC3199523

[GR279105WOMC2] Ai S, Xiong H, Li CC, Luo Y, Shi Q, Liu Y, Yu X, Li C, He A. 2019. Profiling chromatin states using single-cell itChIP-seq. Nat Cell Biol 21: 1164–1172. 10.1038/s41556-019-0383-5 31481796

[GR279105WOMC3] Amemiya HM, Kundaje A, Boyle AP. 2019. The ENCODE blacklist: identification of problematic regions of the genome. Sci Rep 9: 9354. 10.1038/s41598-019-45839-z 31249361 PMC6597582

[GR279105WOMC4] Ashrafizadeh M, Zarrabi A, Hushmandi K, Kalantari M, Mohammadinejad R, Javaheri T, Sethi G. 2020. Association of the epithelial–mesenchymal transition (EMT) with cisplatin resistance. Int J Mol Sci 21: 4002. 10.3390/ijms21114002 32503307 PMC7312011

[GR279105WOMC5] Brock A, Chang H, Huang S. 2009. Non-genetic heterogeneity—a mutation-independent driving force for the somatic evolution of tumours. Nat Rev Genet 10: 336–342. 10.1038/nrg2556 19337290

[GR279105WOMC6] Brunson JC. 2020. ggalluvial: layered grammar for alluvial plots. J Open Source Softw 5: 2017. 10.21105/joss.02017 36919162 PMC10010671

[GR279105WOMC7] Carter B, Ku WL, Kang JY, Hu G, Perrie J, Tang Q, Zhao K. 2019. Mapping histone modifications in low cell number and single cells using antibody-guided chromatin tagmentation (ACT-seq). Nat Commun 10: 3747. 10.1038/s41467-019-11559-1 31431618 PMC6702168

[GR279105WOMC8] Chia S, Low J-L, Zhang X, Kwang X-L, Chong F-T, Sharma A, Bertrand D, Toh SY, Leong H-S, Thangavelu MT, 2017. Phenotype-driven precision oncology as a guide for clinical decisions one patient at a time. Nat Commun 8: 435. 10.1038/s41467-017-00451-5 28874669 PMC5585361

[GR279105WOMC9] Choi J-H, Lee B-S, Jang JY, Lee YS, Kim HJ, Roh J, Shin YS, Woo HG, Kim C-H. 2023. Single-cell transcriptome profiling of the stepwise progression of head and neck cancer. Nat Commun 14: 1055. 10.1038/s41467-023-36691-x 36828832 PMC9958029

[GR279105WOMC10] Colak S, Ten Dijke P. 2017. Targeting TGF-β signaling in cancer. Trends Cancer 3: 56–71. 10.1016/j.trecan.2016.11.008 28718426

[GR279105WOMC11] Easwaran H, Tsai H-C, Baylin SB. 2014. Cancer epigenetics: tumor heterogeneity, plasticity of stem-like states, and drug resistance. Mol Cell 54: 716–727. 10.1016/j.molcel.2014.05.015 24905005 PMC4103691

[GR279105WOMC12] Ernst J, Kellis M. 2012. ChromHMM: automating chromatin-state discovery and characterization. Nat Methods 9: 215–216. 10.1038/nmeth.1906 22373907 PMC3577932

[GR279105WOMC13] Fornes O, Castro-Mondragon JA, Khan A, van der Lee R, Zhang X, Richmond PA, Modi BP, Correard S, Gheorghe M, Baranašić D, 2020. JASPAR 2020: update of the open-access database of transcription factor binding profiles. Nucleic Acids Res 48: D87–D92. 10.1093/nar/gkz1001 31701148 PMC7145627

[GR279105WOMC14] Fukusumi T, Califano JA. 2018. The *NOTCH* pathway in head and neck squamous cell carcinoma. J Dent Res 97: 645–653. 10.1177/0022034518760297 29489439 PMC5960881

[GR279105WOMC15] Füllgrabe J, Kavanagh E, Joseph B. 2011. Histone onco-modifications. Oncogene 30: 3391–3403. 10.1038/onc.2011.121 21516126

[GR279105WOMC16] Gu Z, Hübschmann D. 2023. *rGREAT*: an R/Bioconductor package for functional enrichment on genomic regions. Bioinformatics 39: btac745. 10.1093/bioinformatics/btac745 36394265 PMC9805586

[GR279105WOMC17] Guo M, Peng Y, Gao A, Du C, Herman JG. 2019. Epigenetic heterogeneity in cancer. Biomark Res 7: 23. 10.1186/s40364-019-0174-y 31695915 PMC6824025

[GR279105WOMC18] Hainer SJ, Bošković A, McCannell KN, Rando OJ, Fazzio TG. 2019. Profiling of pluripotency factors in single cells and early embryos. Cell 177: 1319–1329.e11. 10.1016/j.cell.2019.03.014 30955888 PMC6525046

[GR279105WOMC19] Hamilton NE, Ferry M. 2018. ggtern: ternary diagrams using ggplot2. J Stat Softw 87: 1–17. 10.18637/jss.v087.c03

[GR279105WOMC20] Helin K, Dhanak D. 2013. Chromatin proteins and modifications as drug targets. Nature 502: 480–488. 10.1038/nature12751 24153301

[GR279105WOMC21] Huang D, Savage SR, Calinawan AP, Lin C, Zhang B, Wang P, Starr TK, Birrer MJ, Paulovich AG. 2021. A highly annotated database of genes associated with platinum resistance in cancer. Oncogene 40: 6395–6405. 10.1038/s41388-021-02055-2 34645978 PMC8602037

[GR279105WOMC22] Hyun K, Jeon J, Park K, Kim J. 2017. Writing, erasing and reading histone lysine methylations. Exp Mol Med 49: e324. 10.1038/emm.2017.11 28450737 PMC6130214

[GR279105WOMC23] Kakiuchi A, Kakuki T, Ohwada K, Kurose M, Kondoh A, Obata K, Nomura K, Miyata R, Kaneko Y, Konno T, 2021. HDAC inhibitors suppress the proliferation, migration and invasiveness of human head and neck squamous cell carcinoma cells via p63-mediated tight junction molecules and p21-mediated growth arrest. Oncol Rep 45: 46. 10.3892/or.2021.7997 33649777 PMC7934225

[GR279105WOMC24] Kaya-Okur HS, Wu SJ, Codomo CA, Pledger ES, Bryson TD, Henikoff JG, Ahmad K, Henikoff S. 2019. CUT&tag for efficient epigenomic profiling of small samples and single cells. Nat Commun 10: 1930. 10.1038/s41467-019-09982-5 31036827 PMC6488672

[GR279105WOMC25] Kaya-Okur HS, Janssens DH, Henikoff JG, Ahmad K, Henikoff S. 2020. Efficient low-cost chromatin profiling with CUT&Tag. Nat Protoc 15: 3264–3283. 10.1038/s41596-020-0373-x 32913232 PMC8318778

[GR279105WOMC26] Kim DC, Jin H, Lee JS, Son E, Lee GW, Kim HJ. 2020. P2y2r has a significant correlation with notch-4 in patients with breast cancer. Oncol Lett 20: 647–654. 10.3892/ol.2020.11630 32565989 PMC7286009

[GR279105WOMC27] Lamar JM, Stern P, Liu H, Schindler JW, Jiang Z-G, Hynes RO. 2012. The hippo pathway target, YAP, promotes metastasis through its TEAD-interaction domain. Proc Natl Acad Sci 109: E2441–E2450. 10.1073/pnas.1212021109 22891335 PMC3443162

[GR279105WOMC28] Langmead B, Salzberg SL. 2012. Fast gapped-read alignment with Bowtie 2. Nat Methods 9: 357–359. 10.1038/nmeth.1923 22388286 PMC3322381

[GR279105WOMC29] Li H, Handsaker B, Wysoker A, Fennell T, Ruan J, Homer N, Marth G, Abecasis G, Durbin R, 1000 Genome Project Data Processing Subgroup. 2009. The Sequence Alignment/Map format and SAMtools. Bioinformatics 25: 2078–2079. 10.1093/bioinformatics/btp352 19505943 PMC2723002

[GR279105WOMC30] Li Z, Zhou W, Zhang Y, Sun W, Yung MM, Sun J, Li J, Chen C-W, Li Z, Meng Y, 2019. ERK regulates HIF-1α-mediated platinum resistance by directly targeting PHD2 in ovarian cancer. Clin Cancer Res 25: 5947–5960. 10.1158/1078-0432.CCR-18-4145 31285371 PMC7449248

[GR279105WOMC31] Lin J, Xia L, Oyang L, Liang J, Tan S, Wu N, Yi P, Pan Q, Rao S, Han Y, 2022. The POU2F1-ALDOA axis promotes the proliferation and chemoresistance of colon cancer cells by enhancing glycolysis and the pentose phosphate pathway activity. Oncogene 41: 1024–1039. 10.1038/s41388-021-02148-y 34997215 PMC8837540

[GR279105WOMC32] Litzenburger UM, Buenrostro JD, Wu B, Shen Y, Sheffield NC, Kathiria A, Greenleaf WJ, Chang HY. 2017. Single-cell epigenomic variability reveals functional cancer heterogeneity. Genome Biol 18: 15. 10.1186/s13059-016-1133-7 28118844 PMC5259890

[GR279105WOMC33] Love MI, Huber W, Anders S. 2014. Moderated estimation of fold change and dispersion for RNA-seq data with DESeq2. Genome Biol 15: 550. 10.1186/s13059-014-0550-8 25516281 PMC4302049

[GR279105WOMC34] Love MI, Anders S, Kim V, Huber W. 2019. RNA-seq workflow: gene-level exploratory analysis and differential expression. F1000Res 4: 1070. 10.12688/f1000research.7035.1 PMC4670015 26674615

[GR279105WOMC35] Love MI, Soneson C, Hickey PF, Johnson LK, Pierce NT, Shepherd L, Morgan M, Patro R. 2020. Tximeta: reference sequence checksums for provenance identification in RNA-seq. PLoS Comput Biol 16: e1007664. 10.1371/journal.pcbi.1007664 32097405 PMC7059966

[GR279105WOMC36] Matthias C, Mack B, Berghaus A, Gires O. 2008. Keratin 8 expression in head and neck epithelia. BMC Cancer 8: 267. 10.1186/1471-2407-8-267 18803884 PMC2556347

[GR279105WOMC37] McKenna A, Hanna M, Banks E, Sivachenko A, Cibulskis K, Kernytsky A, Garimella K, Altshuler D, Gabriel S, Daly M, 2010. The genome analysis toolkit: a MapReduce framework for analyzing next-generation DNA sequencing data. Genome Res 20: 1297–1303. 10.1101/gr.107524.110 20644199 PMC2928508

[GR279105WOMC38] McLean CY, Bristor D, Hiller M, Clarke SL, Schaar BT, Lowe CB, Wenger AM, Bejerano G. 2010. GREAT improves functional interpretation of *cis*-regulatory regions. Nat Biotechnol 28: 495–501. 10.1038/nbt.1630 20436461 PMC4840234

[GR279105WOMC39] Meers MP, Bryson TD, Henikoff JG, Henikoff S. 2019. Improved CUT&RUN chromatin profiling tools. eLife 8: e46314. 10.7554/eLife.46314 31232687 PMC6598765

[GR279105WOMC40] Minussi DC, Nicholson MD, Ye H, Davis A, Wang K, Baker T, Tarabichi M, Sei E, Du H, Rabbani M, 2021. Breast tumours maintain a reservoir of subclonal diversity during expansion. Nature 592: 302–308. 10.1038/s41586-021-03357-x 33762732 PMC8049101

[GR279105WOMC41] Morton AR, Dogan-Artun N, Faber ZJ, MacLeod G, Bartels CF, Piazza MS, Allan KC, Mack SC, Wang X, Gimple RC, 2019. Functional enhancers shape extrachromosomal oncogene amplifications. Cell 179: 1330–1341.e13. 10.1016/j.cell.2019.10.039 31761532 PMC7241652

[GR279105WOMC42] Omori H, Nishio M, Masuda M, Miyachi Y, Ueda F, Nakano T, Sato K, Mimori K, Taguchi K, Hikasa H, 2020. YAP1 is a potent driver of the onset and progression of oral squamous cell carcinoma. Sci Adv 6: eaay3324. 10.1126/sciadv.aay3324 32206709 PMC7080500

[GR279105WOMC43] Patro R, Duggal G, Love MI, Irizarry RA, Kingsford C. 2017. Salmon provides fast and bias-aware quantification of transcript expression. Nat Methods 14: 417–419. 10.1038/nmeth.4197 28263959 PMC5600148

[GR279105WOMC44] Patty BJ, Hainer SJ. 2021. Transcription factor chromatin profiling genome-wide using uliCUT&RUN in single cells and individual blastocysts. Nat Protoc 16: 2633–2666. 10.1038/s41596-021-00516-2 33911257 PMC8177051

[GR279105WOMC45] Pisco AO, Huang S. 2015. Non-genetic cancer cell plasticity and therapy-induced stemness in tumour relapse: ‘What does not kill me strengthens me.’ Br J Cancer 112: 1725–1732. 10.1038/bjc.2015.146 25965164 PMC4647245

[GR279105WOMC46] Puram SV, Tirosh I, Parikh AS, Patel AP, Yizhak K, Gillespie S, Rodman C, Luo CL, Mroz EA, Emerick KS, 2017. Single-cell transcriptomic analysis of primary and metastatic tumor ecosystems in head and neck cancer. Cell 171: 1611–1624.e24. 10.1016/j.cell.2017.10.044 29198524 PMC5878932

[GR279105WOMC47] Qi Z, Liu Y, Mints M, Mullins R, Sample R, Law T, Barrett T, Mazul AL, Jackson RS, Kang SY, 2021. Single-cell deconvolution of head and neck squamous cell carcinoma. Cancers (Basel) 13: 1230. 10.3390/cancers13061230 33799782 PMC7999850

[GR279105WOMC48] Qu J, Tanis SEJ, Smits JPH, Kouwenhoven EN, Oti M, van den Bogaard EH, Logie C, Stunnenberg HG, van Bokhoven H, Mulder KW, 2018. Mutant p63 affects epidermal cell identity through rewiring the enhancer landscape. Cell Rep 25: 3490–3503.e4. 10.1016/j.celrep.2018.11.039 30566872

[GR279105WOMC49] Quah HS, Cao EY, Suteja L, Li CH, Leong HS, Chong FT, Gupta S, Arcinas C, Ouyang JF, Ang V, 2023. Single cell analysis in head and neck cancer reveals potential immune evasion mechanisms during early metastasis. Nat Commun 14: 1680. 10.1038/s41467-023-37379-y 36973261 PMC10042873

[GR279105WOMC50] Quinlan AR, Hall IM. 2010. BEDTools: a flexible suite of utilities for comparing genomic features. Bioinformatics 26: 841–842. 10.1093/bioinformatics/btq033 20110278 PMC2832824

[GR279105WOMC51] R Core Team. 2024. R: a language and environment for statistical computing. R Foundation for Statistical Computing, Vienna. https://www.R-project.org/.

[GR279105WOMC52a] Robinson JT, Thorvaldsdóttir H, Winckler W, Guttman M, Lander ES, Getz G, Mesirov JP. 2011. Integrative genomics viewer. Nat Biotechnol 29: 24–26. 10.1038/nbt.1754 21221095 PMC3346182

[GR279105WOMC52] Rotem A, Ram O, Shoresh N, Sperling RA, Goren A, Weitz DA, Bernstein BE. 2015. Single-cell ChIP-seq reveals cell subpopulations defined by chromatin state. Nat Biotechnol 33: 1165–1172. 10.1038/nbt.3383 26458175 PMC4636926

[GR279105WOMC53] Ruiz-Villalba A, van Pelt-Verkuil E, Gunst QD, Ruijter JM, van den Hoff MJ. 2017. Amplification of nonspecific products in quantitative polymerase chain reactions (qPCR). Biomol Detect Quantif 14: 7–18. 10.1016/j.bdq.2017.10.001 29255685 PMC5727009

[GR279105WOMC53a] Schep AN, Wu B, Buenrostro JD, Greenleaf WJ. 2017. chromVAR: inferring transcription-factor-associated accessibility from single-cell epigenomic data. Nat Methods 14: 975–978. 10.1038/nmeth.4401 28825706 PMC5623146

[GR279105WOMC54] Segre JA, Bauer C, Fuchs E. 1999. Klf4 is a transcription factor required for establishing the barrier function of the skin. Nat Genet 22: 356–360. 10.1038/11926 10431239

[GR279105WOMC55] Sharma A, Cao EY, Kumar V, Zhang X, Leong HS, Wong AML, Ramakrishnan N, Hakimullah M, Teo HMV, Chong FT, 2018. Longitudinal single-cell RNA sequencing of patient-derived primary cells reveals drug-induced infidelity in stem cell hierarchy. Nat Commun 9: 4931. 10.1038/s41467-018-07261-3 30467425 PMC6250721

[GR279105WOMC56] Shin E, Kim J. 2020. The potential role of YAP in head and neck squamous cell carcinoma. Exp Mol Med 52: 1264–1274. 10.1038/s12276-020-00492-9 32859951 PMC8080831

[GR279105WOMC57] Skene PJ, Henikoff S. 2017. An efficient targeted nuclease strategy for high-resolution mapping of DNA binding sites. eLife 16: e21856. 10.7554/eLife.21856 PMC5310842 28079019

[GR279105WOMC58] Skene PJ, Henikoff JG, Henikoff S. 2018. Targeted *in situ* genome-wide profiling with high efficiency for low cell numbers. Nat Protoc 13: 1006–1019. 10.1038/nprot.2018.015 29651053

[GR279105WOMC59] Stuart T, Butler A, Hoffman P, Hafemeister C, Papalexi E, Mauck WM, Hao Y, Stoeckius M, Smibert P, Satija R. 2019. Comprehensive integration of single-cell data. Cell 177: 1888–1902.e21. 10.1016/j.cell.2019.05.031 31178118 PMC6687398

[GR279105WOMC60] Stuart T, Srivastava A, Madad S, Lareau CA, Satija R. 2021. Single-cell chromatin state analysis with Signac. Nat Methods 18: 1333–1341. 10.1038/s41592-021-01282-5 34725479 PMC9255697

[GR279105WOMC61] Talevich E, Shain AH, Botton T, Bastian BC. 2016. CNVkit: genome-wide copy number detection and visualization from targeted DNA sequencing. PLoS Comput Biol 12: e1004873. 10.1371/journal.pcbi.1004873 27100738 PMC4839673

[GR279105WOMC62] Valencia-Sama I, Zhao Y, Lai D, Janse van Rensburg HJ, Hao Y, Yang X. 2015. Hippo component TAZ functions as a co-repressor and negatively regulates *ΔNp63* transcription through TEA domain (TEAD) transcription factor. J Biol Chem 290: 16906–16917. 10.1074/jbc.M115.642363 25995450 PMC4505436

[GR279105WOMC63] Vázquez-Arreguín K, Bensard C, Schell JC, Swanson E, Chen X, Rutter J, Tantin D. 2019. Oct1/Pou2f1 is selectively required for colon regeneration and regulates colon malignancy. PLoS Genet 15: e1007687. 10.1371/journal.pgen.1007687 31059499 PMC6522070

[GR279105WOMC64] Wang R, Xin M, Li Y, Zhang P, Zhang M. 2016. The functions of histone modification enzymes in cancer. CPPS 17: 438–445. 10.2174/1389203717666160122120521 26796305

[GR279105WOMC65] Wang Q, Xiong H, Ai S, Yu X, Liu Y, Zhang J, He A. 2019. CoBATCH for high-throughput single-cell epigenomic profiling. Mol Cell 76: 206–216.e7. 10.1016/j.molcel.2019.07.015 31471188

[GR279105WOMC66] Wang Y, Dong C, Zhou BP. 2020. Metabolic reprogram associated with epithelial-mesenchymal transition in tumor progression and metastasis. Genes Dis 7: 172–184. 10.1016/j.gendis.2019.09.012 32215287 PMC7083713

[GR279105WOMC67] Woods LT, Jasmer KJ, Muñoz Forti K, Shanbhag VC, Camden JM, Erb L, Petris MJ, Weisman GA. 2020. P2Y_2_ receptors mediate nucleotide-induced EGFR phosphorylation and stimulate proliferation and tumorigenesis of head and neck squamous cell carcinoma cell lines. Oral Oncol 109: 104808. 10.1016/j.oraloncology.2020.104808 32540611 PMC7736485

[GR279105WOMC68] Wu S, Turner KM, Nguyen N, Raviram R, Erb M, Santini J, Luebeck J, Rajkumar U, Diao Y, Li B, 2019. Circular ecDNA promotes accessible chromatin and high oncogene expression. Nature 575: 699–703. 10.1038/s41586-019-1763-5 31748743 PMC7094777

[GR279105WOMC69] Xie Y, Zhu C, Wang Z, Tastemel M, Chang L, Li YE, Ren B. 2023. Droplet-based single-cell joint profiling of histone modifications and transcriptomes. Nat Struct Mol Biol 30: 1428–1433. 10.1038/s41594-023-01060-1 37563440 PMC10584685

[GR279105WOMC70] Xiong H, Luo Y, Wang Q, Yu X, He A. 2021. Single-cell joint detection of chromatin occupancy and transcriptome enables higher-dimensional epigenomic reconstructions. Nat Methods 18: 652–660. 10.1038/s41592-021-01129-z 33958790

[GR279105WOMC71] Yu G, Wang L-G, He Q-Y. 2015. ChIPseeker: an R/Bioconductor package for ChIP peak annotation, comparison and visualization. Bioinformatics 31: 2382–2383. 10.1093/bioinformatics/btv145 25765347

[GR279105WOMC72] Yu W, Chen Y, Putluri N, Coarfa C, Robertson MJ, Putluri V, Stossi F, Dubrulle J, Mancini MA, Pang JC, 2020. Acquisition of cisplatin resistance shifts head and neck squamous cell carcinoma metabolism toward neutralization of oxidative stress. Cancers (Basel) 12: 1670. 10.3390/cancers12061670 32599707 PMC7352569

[GR279105WOMC72a] Zhang Y, Liu T, Meyer CA, Eeckhoute J, Johnson DS, Bernstein BE, Nusbaum C, Myers RM, Brown M, Li W, 2008. Model-based Analysis of ChIP-Seq (MACS). Genome Biol 9: R137. 10.1186/gb-2008-9-9-r137 18798982 PMC2592715

[GR279105WOMC73] Zhao X, Mai Z, Liu L, Lu Y, Cui L, Yu J. 2024. Hypoxia-driven TNS4 fosters HNSCC tumorigenesis by stabilizing integrin α5β1 complex and triggering FAK-mediated Akt and TGFβ signaling pathways. Int J Biol Sci 20: 231–248. 10.7150/ijbs.86317 38164166 PMC10750279

[GR279105WOMC74] Zhou Q, Liu S, Kou Y, Yang P, Liu H, Hasegawa T, Su R, Zhu G, Li M. 2022. ATP promotes oral squamous cell carcinoma cell invasion and migration by activating the PI3K/AKT pathway via the P2Y2-Src-EGFR axis. ACS Omega 7: 39760–39771. 10.1021/acsomega.2c03727 36385800 PMC9648055

