# Supplementary material for: Single-nucleus CUT&RUN elucidates the function of intrinsic and genomics-driven epigenetic heterogeneity in head and neck cancer progression
Source: Genome Res. 2025 Jan;35(1):162–77. doi: 10.1101/gr.279105.124 (PMC11789629; doi:10.1101/gr.279105.124)
Supplement: Supplement 3 [file Supplemental_Figures.pdf]

Supplementary Figures

Single-nucleus CUT&RUN deciphers the function of intrinsic and genomics-driven epigenetic heterogeneity in head and neck cancer progression

Howard J. Womersley, Daniel Muliaditan, Ramanuj DasGupta*, Lih Feng Cheow*

*Corresponding authors. Email: dasguptar@gis.a-star.edu.sg (RD), bieclf@nus.edu.sg (LFC)

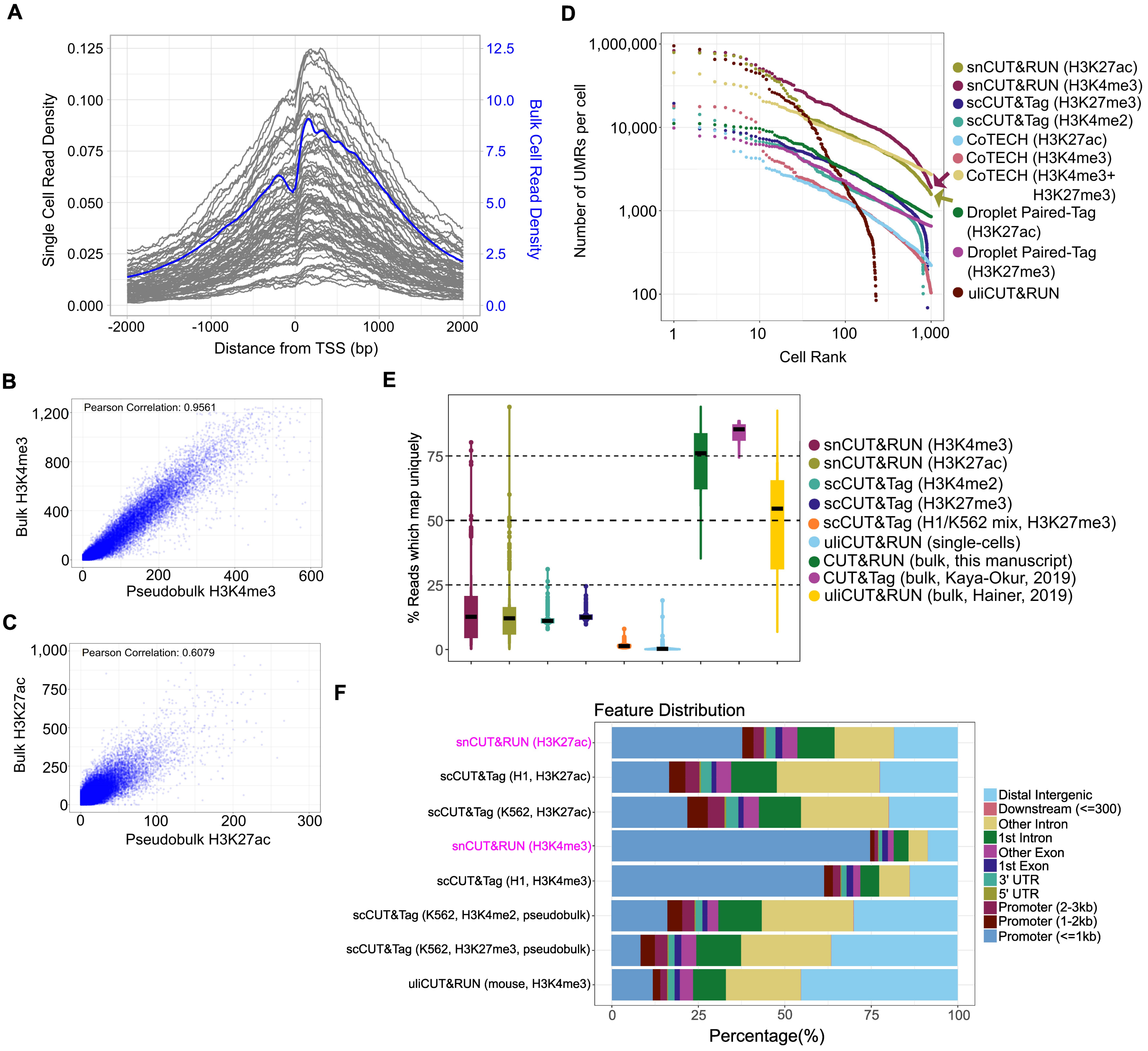
A
Single Cell Read Density
Bulk Cell Read Density
Distance from TSS (bp)
0.125
0.100
0.075
0.050
0.025
0.000
12.5
10.0
7.5
5.0
2.5
0.0
-2000
-1000
0
1000
2000
B
Pearson Correlation: 0.9561
Bulk H3K4me3
Pseudobulk H3K4me3
1,200
800
400
0
200
400
600
C
Pearson Correlation: 0.6079
Bulk H3K27ac
Pseudobulk H3K27ac
1,000
750
500
250
0
100
200
300
D
Number of UMRs per cell
Cell Rank
1,000,000
10,000
1,000
100
1
10
100
1,000
snCUT&RUN (H3K27ac)
snCUT&RUN (H3K4me3)
scCUT&Tag (H3K27me3)
scCUT&Tag (H3K4me2)
CoTECH (H3K27ac)
CoTECH (H3K4me3)
CoTECH (H3K4me3+ H3K27me3)
Droplet Paired-Tag (H3K27ac)
Droplet Paired-Tag (H3K27me3)
uliCUT&RUN
E
% Reads which map uniquely
75
50
25
0
snCUT&RUN (H3K4me3)
snCUT&RUN (H3K27ac)
scCUT&Tag (H3K4me2)
scCUT&Tag (H3K27me3)
scCUT&Tag (H1/K562 mix, H3K27me3)
uliCUT&RUN (single-cells)
CUT&RUN (bulk, this manuscript)
CUT&Tag (bulk, Kaya-Okur, 2019)
uliCUT&RUN (bulk, Hainer, 2019)
F
Feature Distribution
snCUT&RUN (H3K27ac)
scCUT&Tag (H1, H3K27ac)
scCUT&Tag (K562, H3K27ac)
snCUT&RUN (H3K4me3)
scCUT&Tag (H1, H3K4me3)
scCUT&Tag (K562, H3K4me2, pseudobulk)
scCUT&Tag (K562, H3K27me3, pseudobulk)
uliCUT&RUN (mouse, H3K4me3)
0
25
50
75
100
Percentage(%)
Distal Intergenic
Downstream (<=300)
Other Intron
1st Intron
Other Exon
1st Exon
3' UTR
5' UTR
Promoter (2-3kb)
Promoter (1-2kb)
Promoter (<=1kb)

Supplemental Fig. S1. Quality metrics for training and validation data set. **(A)** Comparison of single cell and bulk cell read density for H3K4me3 histone mark relative to global transcriptional start sites. Read density profiles for single cells closely match bulk cell data, with clear nucleosome depletion regions. **(B)** Pearson's correlation between training set single cells and bulk data for H3K4me3 histone mark. **(C)** Pearson's correlation between training set single cells and bulk cell data for H3K27ac histone mark. **(D)** Scatter plot ranking the top 1,000 libraries with the most to the least number of unique mapped reads. snCUT&RUN data is compared with scCUT&Tag (Kaya-Okur et al. 2019), CoTECH (Xiong et al. 2021) and Droplet Paired-Tag (Xie et al. 2023). **(E)** Violin- and boxplots indicating the percentage of reads which map to the genome uniquely after PCR duplicates have been removed. **(F)** ChIPseeker (Yu et al. 2015) annotation of the genomic localization of H3K27ac and H3K4me3 peaks in the snCUT&RUN dataset, compared to the genomic localization of probed histone marks in the scCUT&Tag and uliCUT&RUN bulk/pseudobulk datasets.

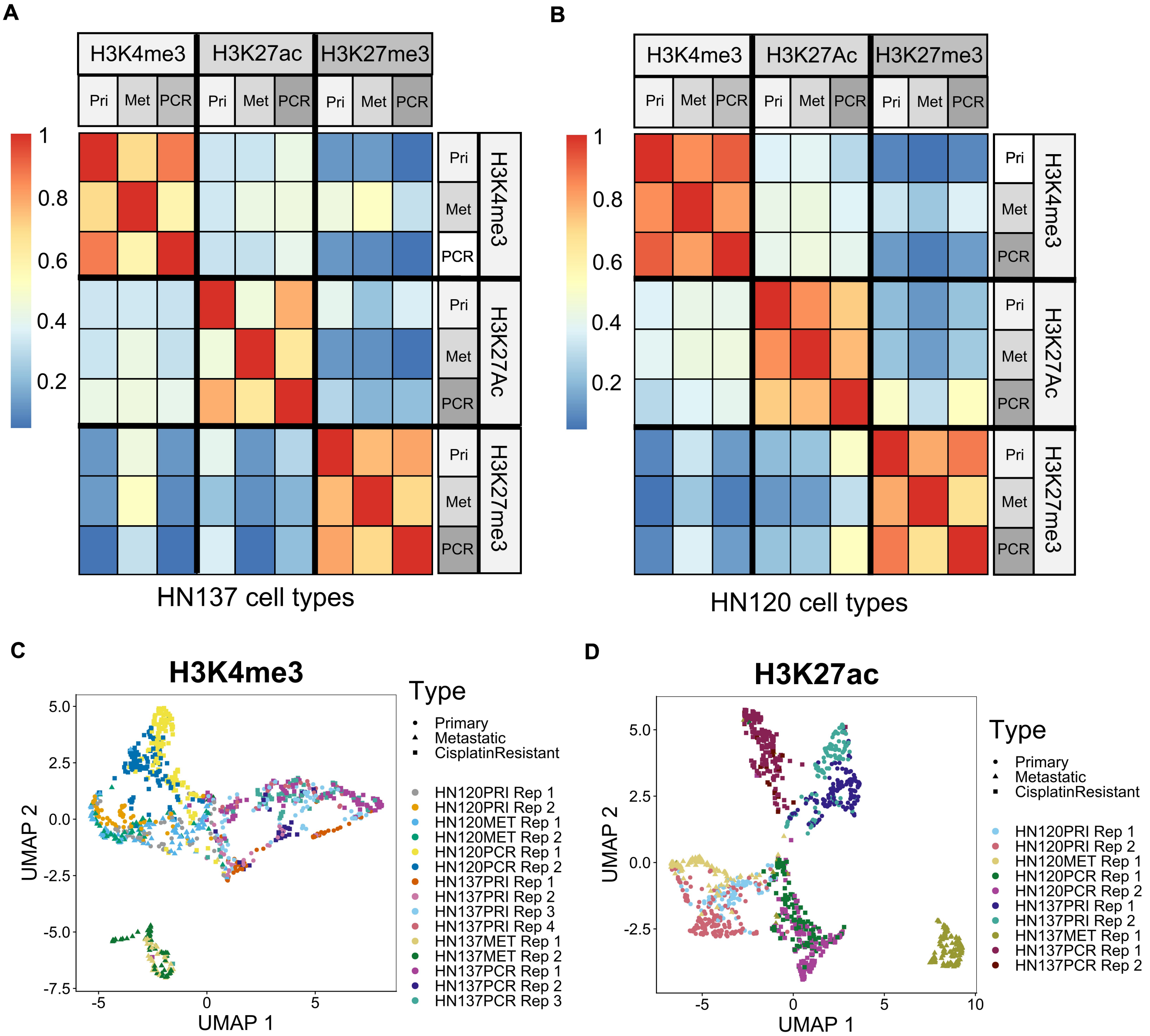

A
H3K4me3
H3K27ac
H3K27me3
Pri
Met
PCR
1
0.8
0.6
0.4
0.2
H3K27Ac
HN137 cell types
B
H3K27Ac
HN120 cell types
C
H3K4me3
UMAP 1
UMAP 2
Type
Primary
Metastatic
CisplatinResistant
HN120PRI Rep 1
HN120PRI Rep 2
HN120MET Rep 1
HN120MET Rep 2
HN120PCR Rep 1
HN120PCR Rep 2
HN137PRI Rep 1
HN137PRI Rep 2
HN137PRI Rep 3
HN137PRI Rep 4
HN137MET Rep 1
HN137MET Rep 2
HN137PCR Rep 1
HN137PCR Rep 2
HN137PCR Rep 3
D
H3K27ac
HN120PRI Rep 1
HN120PRI Rep 2
HN120MET Rep 1
HN120PCR Rep 1
HN120PCR Rep 2
HN137PRI Rep 1
HN137PRI Rep 2
HN137MET Rep 1
HN137PCR Rep 1
HN137PCR Rep 2

Supplemental Fig. S2. Pearson correlation matrices of PRI, MET and PCR cell types for H3K4me3, H3K27ac and H3K27me3 histone marks. **(A)** Similarity matrix for cells from patient HN137. For all histone marks, HN137MET cells have diverged more from HN137PRI than HN137PCR cells. The largest distinction between cell types is between HN137PRI and HN137MET for H3K27ac. **(B)** Similarity matrix for cells from patient HN120. HN120PRI and HN120PCR are more closely associated than HN120MET for both H3K4me3 and H3K27me3 histone marks. For H3K27ac, HN120PCR cells are more distinct from HN120PRI and HN120MET cells. **(C)** and **(D)** UMAPs of H3K4me3 **(C)** and H3K27ac **(D)** profiles of HN120 and HN137 cells annotated by replicates.

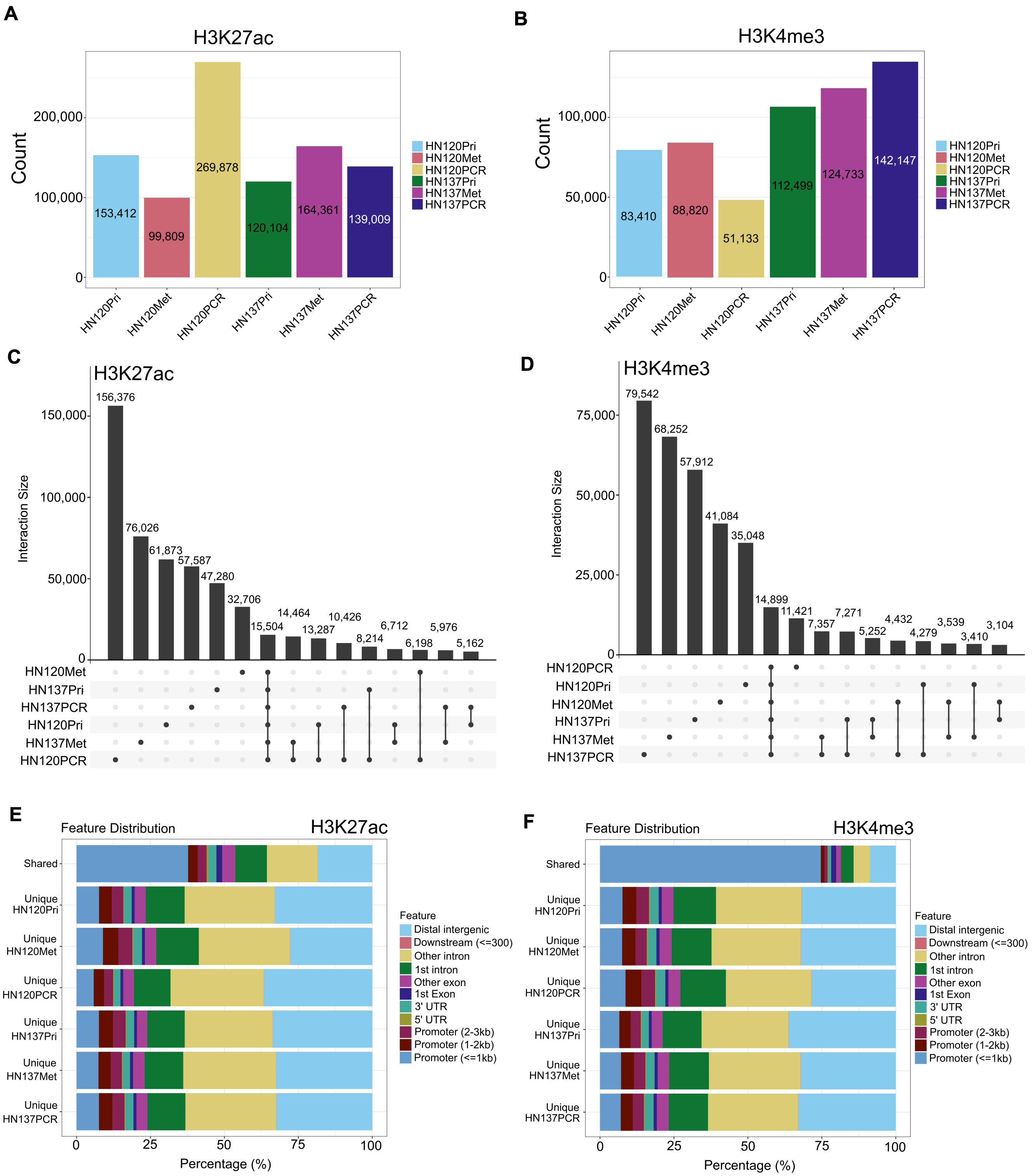

A
H3K27ac
Count
200,000
100,000
0
153,412
99,809
269,878
120,104
164,361
139,009
HN120Pri
HN120Met
HN120PCR
HN137Pri
HN137Met
HN137PCR
B
H3K4me3
Count
100,000
50,000
0
83,410
88,820
51,133
112,499
124,733
142,147
C
H3K27ac
Interaction Size
150,000
100,000
50,000
0
156,376
76,026
61,873
57,587
47,280
32,706
15,504
14,464
13,287
10,426
8,214
6,712
6,198
5,976
5,162
D
H3K4me3
Interaction Size
75,000
50,000
25,000
0
79,542
68,252
57,912
41,084
35,048
14,899
11,421
7,357
7,271
5,252
4,432
4,279
3,539
3,410
3,104
E
Feature Distribution
H3K27ac
Shared
Unique HN120Pri
Unique HN120Met
Unique HN120PCR
Unique HN137Pri
Unique HN137Met
Unique HN137PCR
Percentage (%)
0
25
50
75
100
Feature
Distal intergenic
Downstream (<=300)
Other intron
1st intron
Other exon
1st Exon
3' UTR
5' UTR
Promoter (2-3kb)
Promoter (1-2kb)
Promoter (<=1kb)
F
Feature Distribution
H3K4me3

Supplemental Fig. S3. Global H3K4me3 and H3K27ac profiles of primary and progressed PDCs. (A) and **(B)** Number of H3K27ac **(A)** and H3K4me3 **(B)** peaks achieved per cell line profiled with snCUT&RUN. **(C)** and **(D)** snCUT&RUN number of H3K27ac **(C)** and H3K4me3 **(D)** peaks that are either cell line specific or shared by two or more cell lines. **(E)** and **(F)** ChIPseeker (Yu et al. 2015) annotation of the genomic localization of shared and sample unique H3K27ac **(E)** and H3K4me3 **(F)** peaks in the snCUT&RUN dataset.

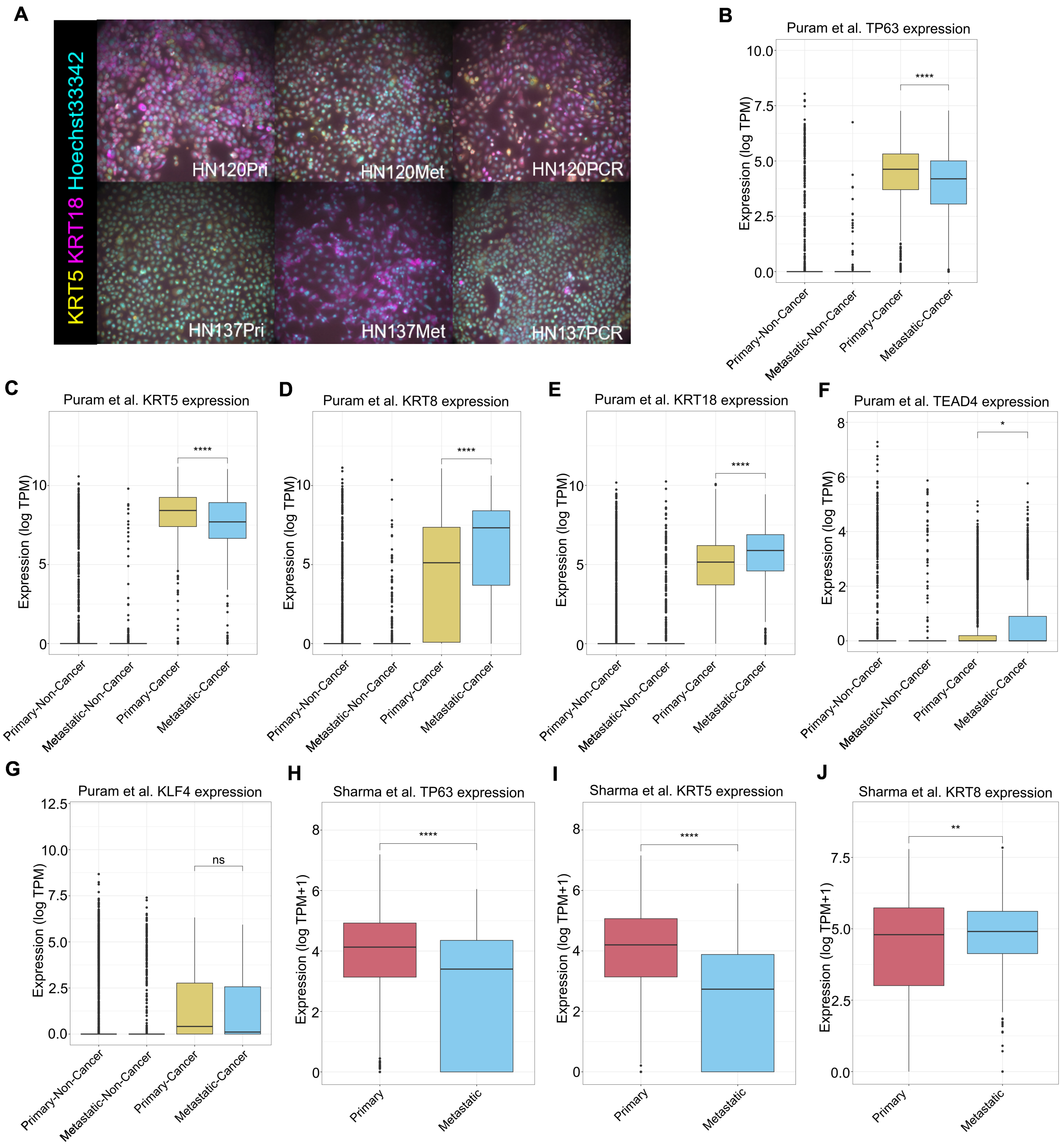

A
KRT5 KRT18 Hoechst33342
HN120Pri
HN120Met
HN120PCR
HN137Pri
HN137Met
HN137PCR
B
Puram et al. TP63 expression
C
Puram et al. KRT5 expression
D
Puram et al. KRT8 expression
E
Puram et al. KRT18 expression
F
Puram et al. TEAD4 expression
G
Puram et al. KLF4 expression
H
Sharma et al. TP63 expression
I
Sharma et al. KRT5 expression
J
Sharma et al. KRT8 expression
Expression (log TPM)
Expression (log TPM+1)
Primary-Non-Cancer
Metastatic-Non-Cancer
Primary-Cancer
Metastatic-Cancer
Primary
Metastatic
****
*
**
ns

Supplemental Fig. S4. Differential keratin expression between primary and progressed HNSCC

(A) Immunofluorescence image of KRT5 (yellow) and KRT18 (magenta) in HN120 and HN137 PDCs. **(B-G)** Expression data from Puram et al. (Puram et al. Cell, 2017) of various keratins and key transcription factors in keratinocyte differentiation, comparing primary tumours and metastatic tumours. **(H-J)** Gene expression of *TP63*, *KRT5* and *KRT8*, comparing primary tumour and metastatic tumours. Data from Sharma et al. Nature Communications, 2018.

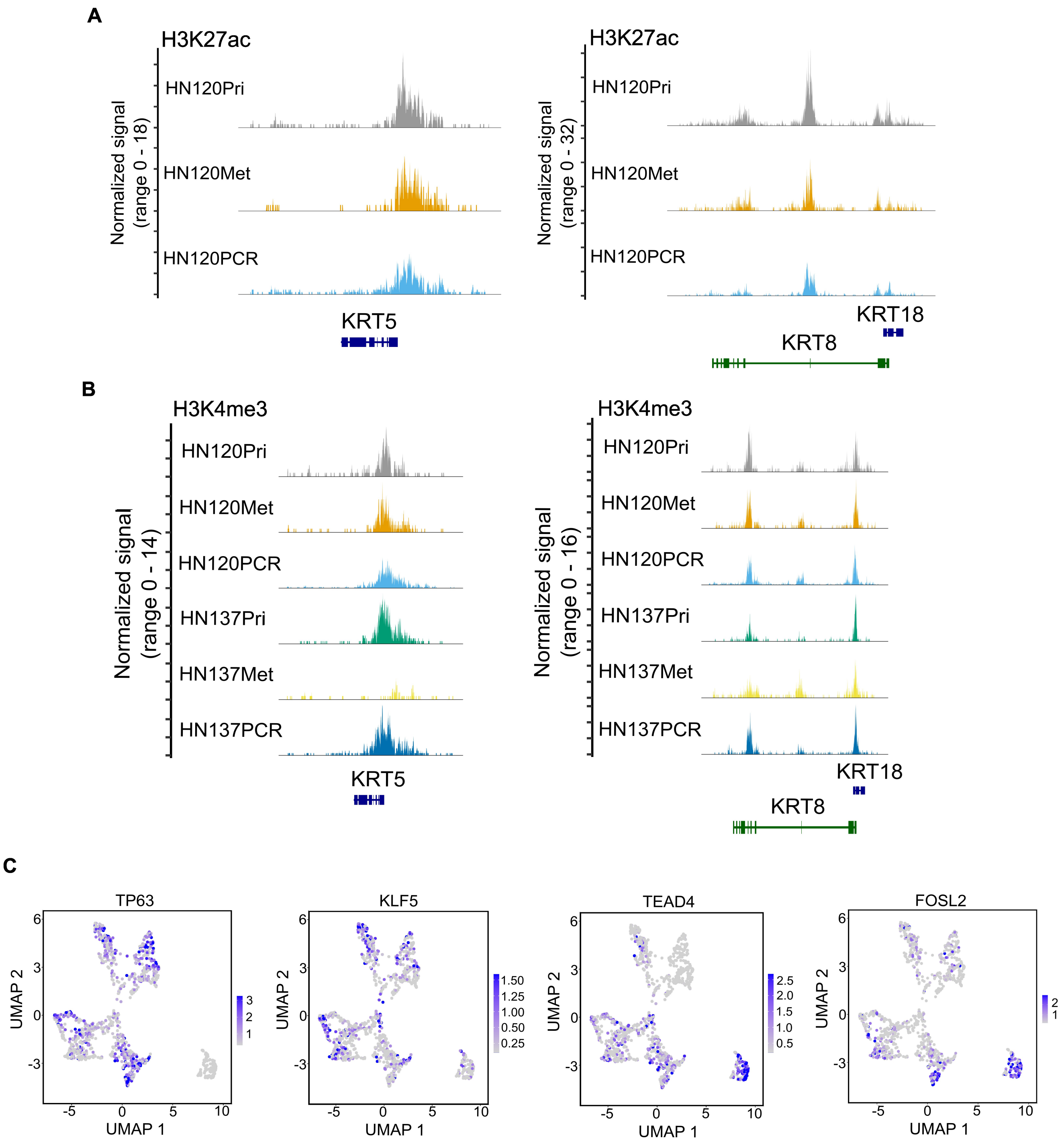
A
H3K27ac
Normalized signal
(range 0 - 18)
HN120Pri
HN120Met
HN120PCR
KRT5
H3K27ac
Normalized signal
(range 0 - 32)
HN120Pri
HN120Met
HN120PCR
KRT18
KRT8
B
H3K4me3
Normalized signal
(range 0 - 14)
HN120Pri
HN120Met
HN120PCR
HN137Pri
HN137Met
HN137PCR
KRT5
H3K4me3
Normalized signal
(range 0 - 16)
HN120Pri
HN120Met
HN120PCR
HN137Pri
HN137Met
HN137PCR
KRT18
KRT8
C
TP63
KLF5
TEAD4
FOSL2
UMAP 2
UMAP 1
6
3
0
-3
-5
0
5
10
3
2
1
1.50
1.25
1.00
0.50
0.25
2.5
2.0
1.5
1.0
0.5
2
1

Supplemental Fig. S5. Inference of transcription factor activity determining epithelial cell identity with H3K27ac and H3K4me3 signal. (A) H3K27ac CoveragePlots at the *KRT5* (left) and *KRT8/18* (right) loci for HN120 PDCs. **(B)** H3K4me3 CoveragePlots for HN120 and HN137 PDCs at the *KRT5* (left) and the *KRT8/18* (right) loci. **(C)** Motif activity UMAPs depicting the TF activities of TP63, KLF5, TEAD4 and FOSL2 at single cell level.

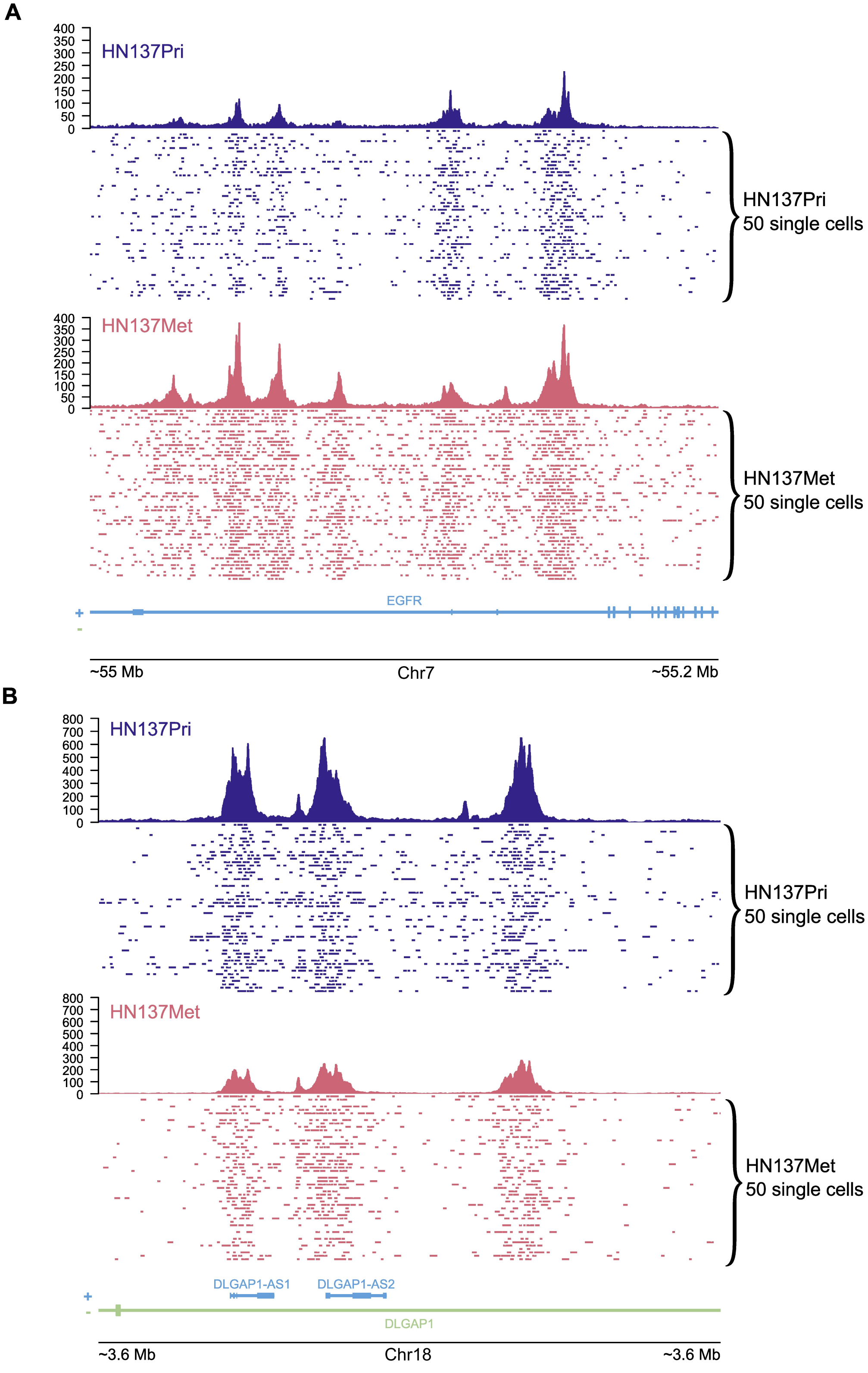
A
HN137Pri
HN137Pri
50 single cells
HN137Met
HN137Met
50 single cells
EGFR
~55 Mb
Chr7
~55.2 Mb
B
HN137Pri
HN137Pri
50 single cells
HN137Met
HN137Met
50 single cells
DLGAP1-AS1
DLGAP1-AS2
DLGAP1
~3.6 Mb
Chr18
~3.6 Mb

Supplemental Fig. S6. Genomic regions with copy number amplifications exhibit intense read density with snCUT&RUN. (A) *EGFR* locus has a higher read density in HN137Met cells than HN137Pri cells, suggesting further amplification of this region might drive primary cancer cells towards metastasis. **(B)** Read density at Chr18p11.31 is significantly higher in both HN137Pri and HN137Met cells compared to the rest of the genome.

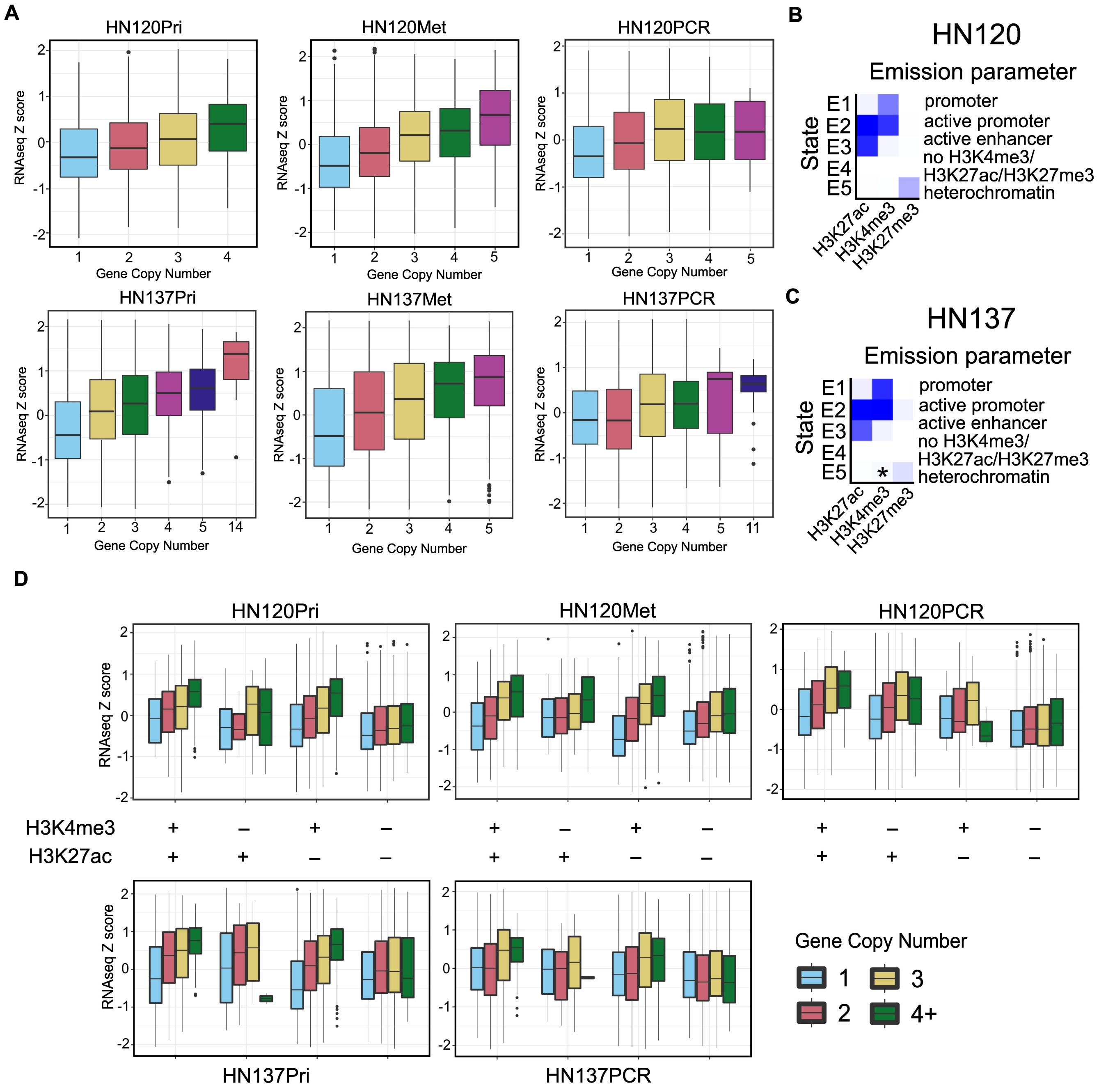
A
HN120Pri
HN120Met
HN120PCR
HN137Pri
HN137Met
HN137PCR
RNAseq Z score
Gene Copy Number
B
HN120
Emission parameter
State
E1
E2
E3
E4
E5
promoter
active promoter
active enhancer
no H3K4me3/
H3K27ac/H3K27me3
heterochromatin
H3K27ac
H3K4me3
H3K27me3
C
HN137
Emission parameter
D
H3K4me3
H3K27ac
Gene Copy Number
1
2
3
4+

Supplemental Fig. S7. Correlation between gene CN, chromatin state, and gene expression (A) Boxplots of RNAseq Z-scores against gene CN of the six PDCs. **(B)** ChromHMM results showing chromatin state combinations based on HN120 samples alone. **(C)** ChromHMM results showing chromatin state combinations based on HN137 samples alone. The weak bivalent H3K4me3/H3K27me3 signal is annotated with an asterisk. **(D)** Boxplots correlating RNAseq Z-scores against H3K27ac/H3K4me3 activity in HN120Pri, HN120Met, HN120PCR, HN137Pri and HN137PCR.

A

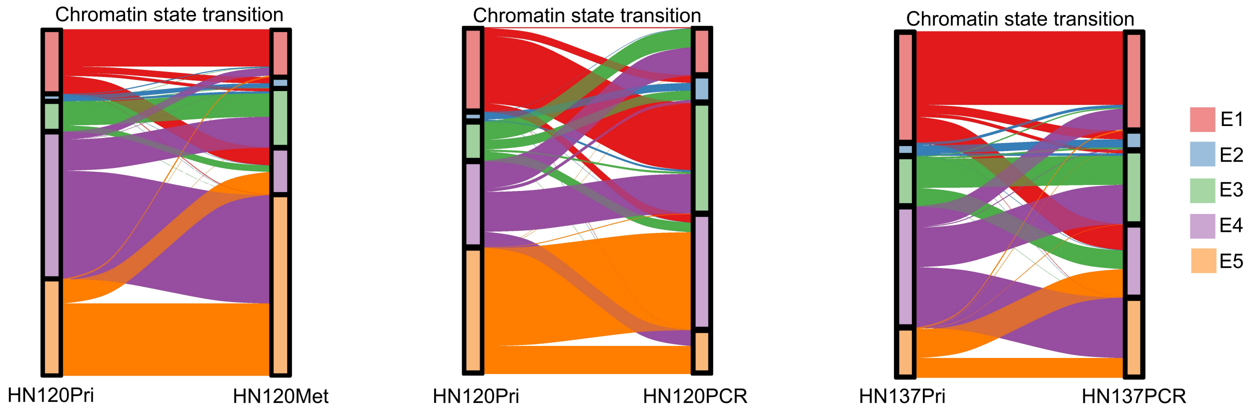

Chromatin state transition
HN120Pri
HN120Met
Chromatin state transition
HN120Pri
HN120PCR
Chromatin state transition
HN137Pri
HN137PCR
E1
E2
E3
E4
E5

B

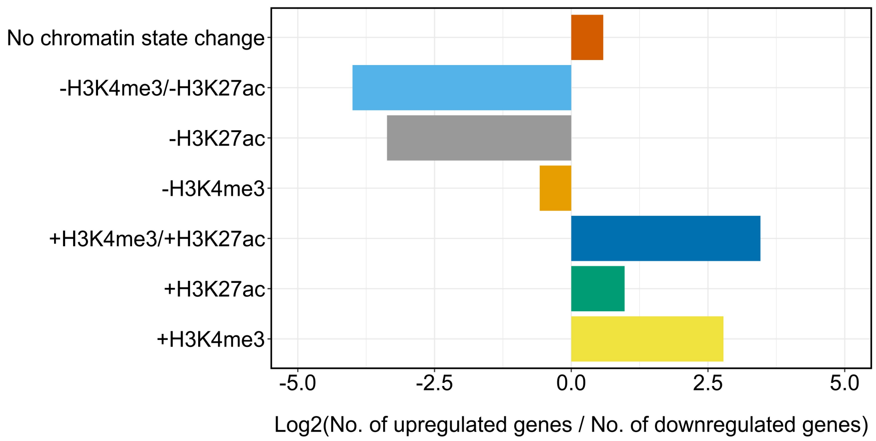

No chromatin state change
-H3K4me3/-H3K27ac
-H3K27ac
-H3K4me3
+H3K4me3/+H3K27ac
+H3K27ac
+H3K4me3
-5.0
-2.5
0.0
2.5
5.0
Log2(No. of upregulated genes / No. of downregulated genes)

Supplemental Fig. S8. Chromatin state transitions between primary tumour and progressed HNSCC. (A) Alluvial plot depicting the chromatin state changes between HN120Pri and HN120Met PDC (left), HN120Pri > HN120PCR transition (middle) and HN137Pri > HN137PCR transition (right). E1 (red): regions with H3K4me3 only. E2 (blue): regions with both H3K4me3 and H3K27ac. E3 (green): regions with H3K27ac only. E4 (purple): unmodified regions. E5 (orange): regions with H3K27me3 only. **(B)** Log2 ratio of the number of upregulated (Log2 FC > 1, $p < 0.001$) genes to the number of downregulated (Log2 FC < -0.5, $p < 0.001$) genes per chromatin state change during metastatic progression of patient HN137 (HN137Pri > HN137Met).

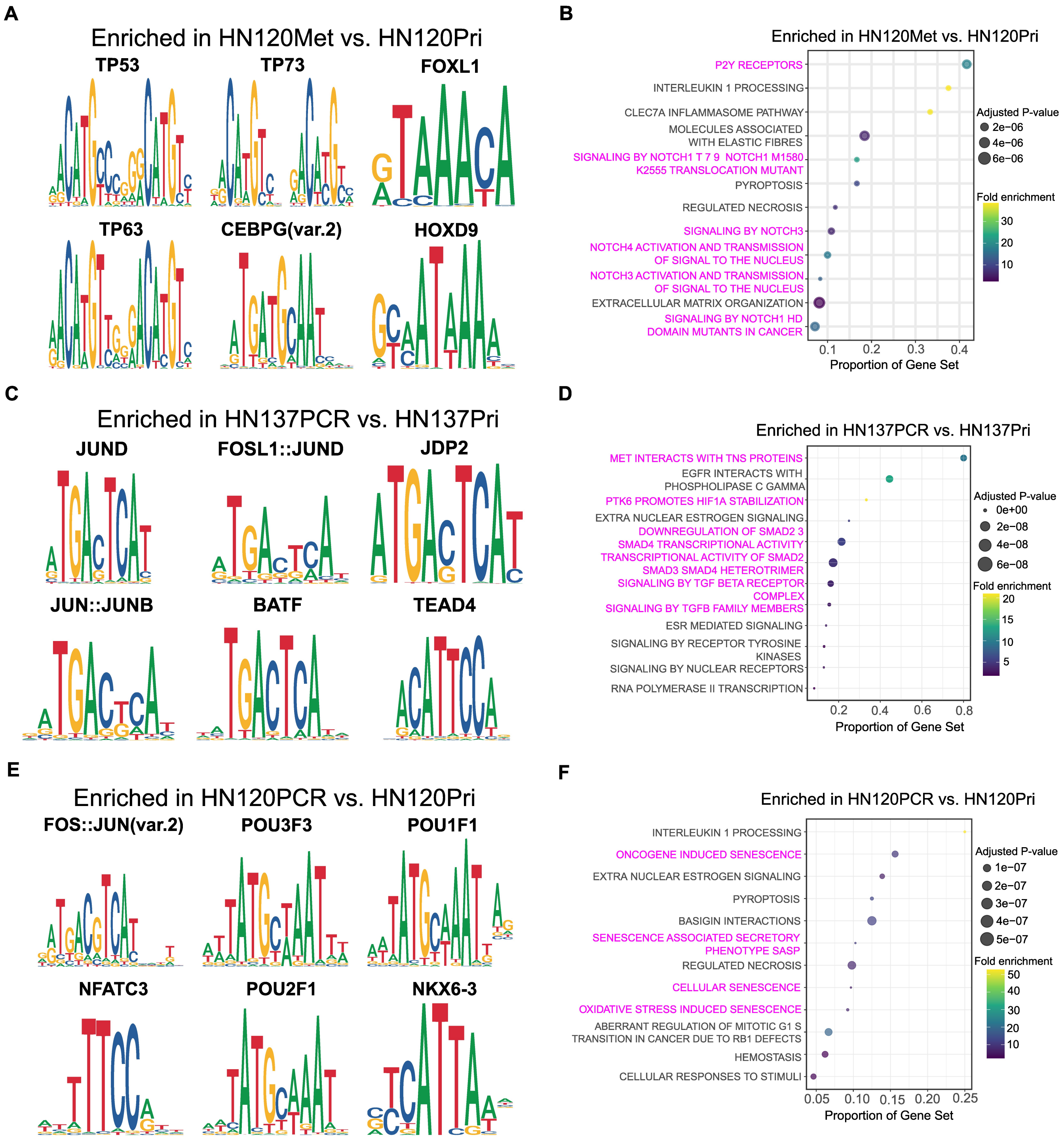
A
Enriched in HN120Met vs. HN120Pri
TP53
TP73
FOXL1
TP63
CEBPG(var.2)
HOXD9
B
Enriched in HN120Met vs. HN120Pri
P2Y RECEPTORS
INTERLEUKIN 1 PROCESSING
CLEC7A INFLAMMASOME PATHWAY
MOLECULES ASSOCIATED WITH ELASTIC FIBRES
SIGNALING BY NOTCH1 T 7 9 NOTCH1 M1580 K2555 TRANSLOCATION MUTANT
PYROPTOSIS
REGULATED NECROSIS
SIGNALING BY NOTCH3
NOTCH4 ACTIVATION AND TRANSMISSION OF SIGNAL TO THE NUCLEUS
NOTCH3 ACTIVATION AND TRANSMISSION OF SIGNAL TO THE NUCLEUS
EXTRACELLULAR MATRIX ORGANIZATION
SIGNALING BY NOTCH1 HD DOMAIN MUTANTS IN CANCER
0.1
0.2
0.3
0.4
Proportion of Gene Set
Adjusted P-value
2e−06
4e−06
6e−06
Fold enrichment
30
20
10
C
Enriched in HN137PCR vs. HN137Pri
JUND
FOSL1::JUND
JDP2
JUN::JUNB
BATF
TEAD4
D
Enriched in HN137PCR vs. HN137Pri
MET INTERACTS WITH TNS PROTEINS
EGFR INTERACTS WITH PHOSPHOLIPASE C GAMMA
PTK6 PROMOTES HIF1A STABILIZATION
EXTRA NUCLEAR ESTROGEN SIGNALING
DOWNREGULATION OF SMAD2 3
SMAD4 TRANSCRIPTIONAL ACTIVITY
TRANSCRIPTIONAL ACTIVITY OF SMAD2
SMAD3 SMAD4 HETEROTRIMER
SIGNALING BY TGF BETA RECEPTOR COMPLEX
SIGNALING BY TGFB FAMILY MEMBERS
ESR MEDIATED SIGNALING
SIGNALING BY RECEPTOR TYROSINE KINASES
SIGNALING BY NUCLEAR RECEPTORS
RNA POLYMERASE II TRANSCRIPTION
0.2
0.4
0.6
0.8
Proportion of Gene Set
Adjusted P-value
0e+00
2e−08
4e−08
6e−08
Fold enrichment
20
15
10
5
E
Enriched in HN120PCR vs. HN120Pri
FOS::JUN(var.2)
POU3F3
POU1F1
NFATC3
POU2F1
NKX6-3
F
Enriched in HN120PCR vs. HN120Pri
INTERLEUKIN 1 PROCESSING
ONCOGENE INDUCED SENESCENCE
EXTRA NUCLEAR ESTROGEN SIGNALING
PYROPTOSIS
BASIGIN INTERACTIONS
SENESCENCE ASSOCIATED SECRETORY PHENOTYPE SASP
REGULATED NECROSIS
CELLULAR SENESCENCE
OXIDATIVE STRESS INDUCED SENESCENCE
ABERRANT REGULATION OF MITOTIC G1 S TRANSITION IN CANCER DUE TO RB1 DEFECTS
HEMOSTASIS
CELLULAR RESPONSES TO STIMULI
0.05
0.10
0.15
0.20
0.25
Proportion of Gene Set
Adjusted P-value
1e−07
2e−07
3e−07
4e−07
5e−07
Fold enrichment
50
40
30
20
10

Supplemental Fig. S9. Transcription factor (TF) activity related to stemness is correlated with HNSCC progression. **(A)** Top six enriched TF motifs in HN120Met vs. HN120Pri. **(B)** Top 12 enriched REACTOME pathways in HN120Met vs. HN120Pri. **(C)** Top six enriched TF motifs in HN137PCR vs. HN137Pri. **(D)** Top 12 enriched REACTOME pathways in HN137PCR vs. HN137Pri. **(E)** Top six enriched TF motifs in HN120PCR vs. HN120Pri. **(F)** Top 12 enriched REACTOME pathways in HN120PCR vs. HN120Pri.

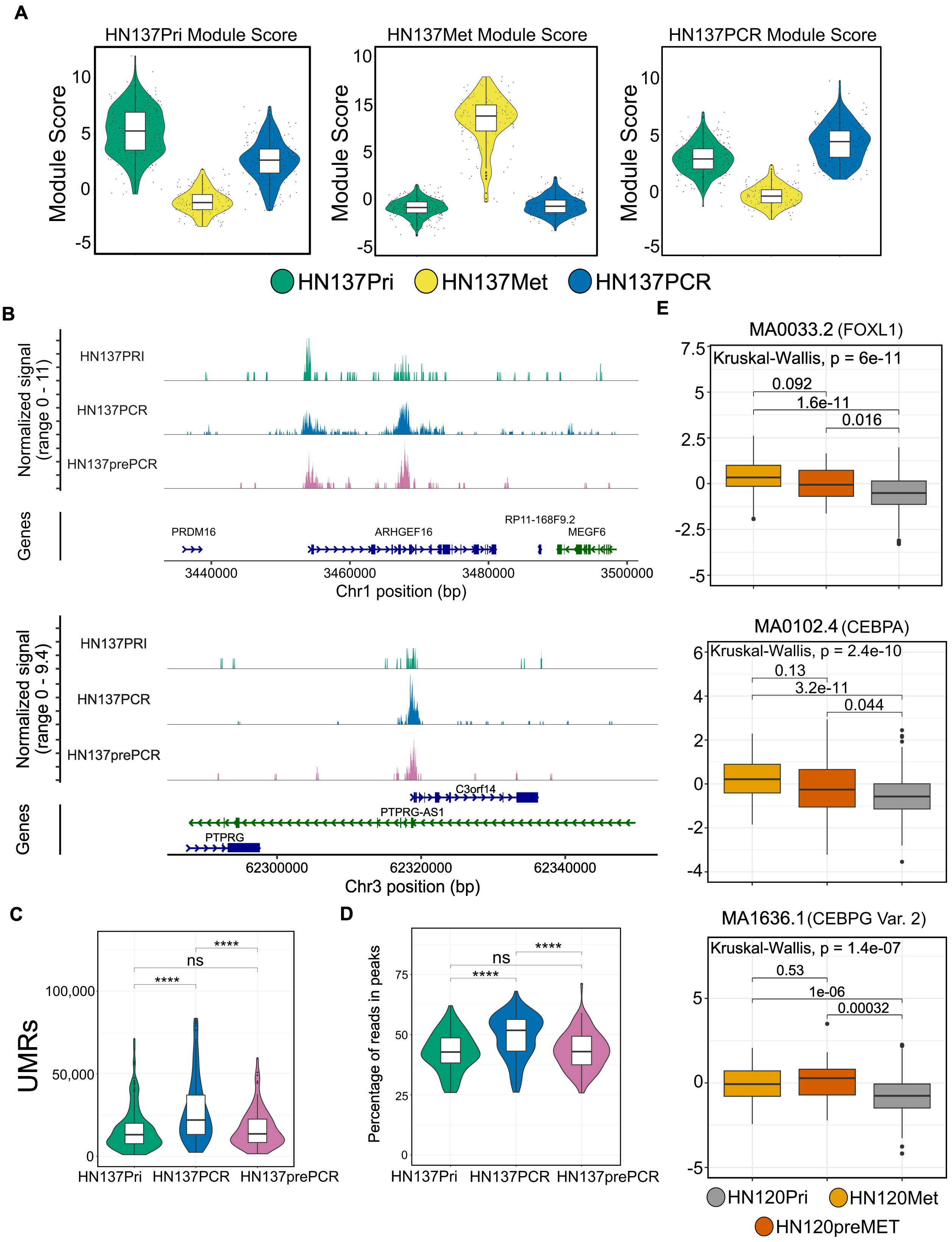

A
HN137Pri Module Score
HN137Met Module Score
HN137PCR Module Score
Module Score
10
5
0
-5
15
HN137Pri
HN137Met
HN137PCR
B
Normalized signal
(range 0 - 11)
HN137PRI
HN137PCR
HN137prePCR
Genes
PRDM16
ARHGEF16
RP11-168F9.2
MEGF6
3440000
3460000
3480000
3500000
Chr1 position (bp)
Normalized signal
(range 0 - 9.4)
C3orf14
PTPRG-AS1
PTPRG
62300000
62320000
62340000
Chr3 position (bp)
C
UMRs
100,000
50,000
****
ns
HN137Pri
HN137PCR
HN137prePCR
D
Percentage of reads in peaks
75
50
25
E
MA0033.2 (FOXL1)
Kruskal-Wallis, p = 6e-11
0.092
1.6e-11
0.016
7.5
2.5
-2.5
MA0102.4 (CEBPA)
Kruskal-Wallis, p = 2.4e-10
0.13
3.2e-11
0.044
MA1636.1 (CEBPG Var. 2)
Kruskal-Wallis, p = 1.4e-07
0.53
1e-06
0.00032
HN120Pri
HN120Met
HN120preMET

Supplemental Fig. S10. HN137prePCR and HN120preMET analysis. (A) PDC-specific HN137 H3K27ac module scores. Each module consists of the top 50 peaks for a particular PDC, and each module score was back calculated for each single-cell using Signac's AddChromatinModule function. **(B)** CoveragePlots of the *ARHGEF16* and *PTPRG-AS1* loci showing higher H3K27ac localization in HN137prePCR compared to remaining HN137Pri cells. **(C)** UMR count for HN137Pri, HN137PCR and HN137prePCR cells. **(D)** FRiP count for HN137Pri, HN137PCR and HN137prePCR cells. **(E)** ChromVAR scores of the activity of several TFs enriched in HN120Met vs. HN120Pri, visualized after the subset of HN120Pri to HN120preMET and remaining HN120Pri cells.
